# An Insight into *Citrus medica* Linn.: A Systematic Review on Phytochemical Profile and Biological Activities

**DOI:** 10.3390/plants12122267

**Published:** 2023-06-10

**Authors:** Nadia Benedetto, Vittorio Carlucci, Immacolata Faraone, Ludovica Lela, Maria Ponticelli, Daniela Russo, Claudia Mangieri, Nikolay T. Tzvetkov, Luigi Milella

**Affiliations:** 1 Department of Science, University of Basilicata, V.le Ateneo Lucano 10, 85100 Potenza, Italy; nadia.benedetto@unibas.it (N.B.); vittorio.carlucci@unibas.it (V.C.); immacolata.faraone@unibas.it (I.F.); ludovica.lela@unibas.it (L.L.); daniela.russo@unibas.it (D.R.); claudia.mangieri@unibas.it (C.M.); 2 Innovative Startup Farmis s.r.l., Via Nicola Vaccaro 40, 85100 Potenza, Italy; 3 Spinoff Bioactiplant, Via dell’Ateneo Lucano 10, 85100 Potenza, Italy; 4 Institute of Molecular Biology “Roumen Tsanev”, Department of Biochemical Pharmacology & Drug Design, Bulgarian Academy of Sciences, Acad. G. Bonchev Str., Bl. 21, 1113 Sofia, Bulgaria; ntzvetkov@gmx.de

**Keywords:** *Citrus medica* Linn., phytochemical composition, biologic effects, systematic review

## Abstract

Plant species are a reservoir of natural compounds that can potentially be used to treat different diseases. *Citrus medica* Linn. belonging to the Rutaceae family, has been used for centuries in medicine for its antioxidant, anti-inflammatory, antimicrobial, antiviral, and antihyperglycemic properties. These activities are ascribable not only to the presence of health-promoting macronutrients and micronutrients, such as carbohydrates, minerals, amino acids, and vitamins, but also to specialized metabolites, such as flavonoids (apigenin, hesperetin, hesperidin, naringin, naringenin, rutin, quercetin, and diosmin), coumarins (citropten, scoparone, and bergapten), terpenes (limonene, *γ*-terpinene, limonin, and nomilin), and phenolic acids (*p*-coumaric acid, trans-ferulic acid, and chlorogenic acid). In recent years, particular attention has been focused on the antioxidant, anti-inflammatory, antimicrobial activity, antidiabetic, anticancer, and neuroprotective activity of *C. medica*. However, although many studies have reported this species’ chemical and biological properties, the literature has never been analyzed via a systematic approach. For this reason, using PubMed and Scopus as databases, we performed a systematic review of *C. medica*’s chemical composition and biological properties to inspire new research approaches and increase its curative application.

## 1. Introduction

**Figure 1:**
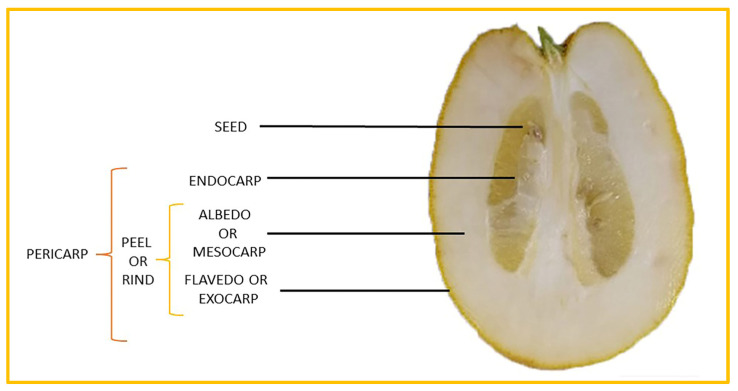
Horizontal cross-section of *C. medica* cultivar, Diamante Liscia, harvested in Italy.

*Citrus medica* Linn., also called “cedar”, “citron”, “etrog”, “foshou”, and “fingered citron”, belonging to the Rutaceae family, is one of the three basic species of the genus *Citrus*, together with *Citrus maxima* Burm. (pomelo) and *Citrus reticulata* Blanco (mandarin). It is a short, medium-sized evergreen tree that reaches 4–8 m in height [1]. Its leaves are up to 20 cm long and its flowers grow in groups of three to twelve. The color of the fruit (size 20–30 cm) varies according to the state of maturation from green to yellow. Anatomically, the *genus Citrus* fruits are composed of exocarp, also called epicarp (flavedo or exterior peel), mesocarp (albedo), and endocarp (locule or segment membrane). Often, the albedo and flavedo are together referred to as the peel or rind. The exocarp or flavedo contains numerous essential oil (EO), glands, carotenoids, and chlorophyll. The mesocarp or white albedo portion of the peel contains cellulose, pectin, and hemicellulose, and it comprises 70% of the fruit, while the endocarp (the edible part of the fruit) and seeds constitute the minor part (Figure 1) [2]. 

**Figure 2:**
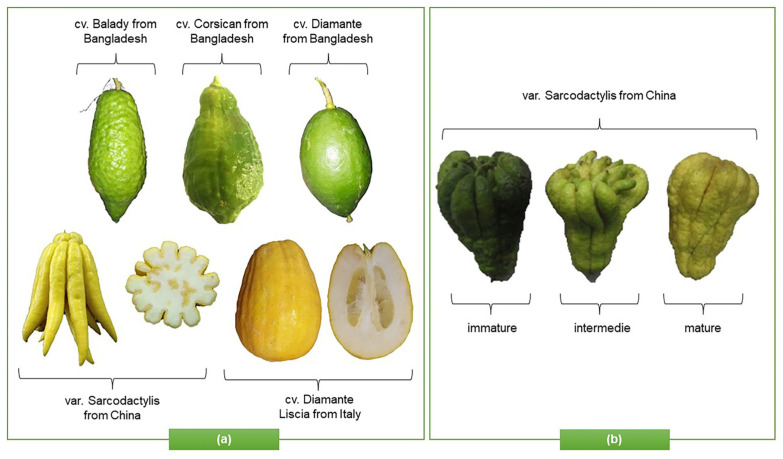
Representation of (**a**) morphological characteristics of some cultivars of *C. medica* from Italy, China, and Bangladesh (**b**) and in different states of maturation.

This species has an ancient origin. It was probably native to Asia Minor before arriving in Europe and, currently, it is widely cultivated in Italy, India, China, Indonesia, Australia, Brazil, and the USA. Furthermore, most citrus fruits prefer a temperate climate, with temperatures of 23–25 °C, and do not tolerate cold below 7–8 °C. “Diamante Liscia”, “Diamante Rugosa”, “Corsican”, “Badaly”, and “Maxima” are the best-known *C. medica* cultivars, while “Sarcodactylis” is the main Chinese variety (var.) (Figure 2a), with different morphological characteristics and phytochemical profiles that depend on the state of maturation (Figure 2b), genetic and agronomic factors, and the habitat [3]. 

Usually, *C. medica* is consumed as a functional food, to prepare beverages, and for medicinal purposes [4]. Described by several botanists, such as Pliny and Theophrasty, due to its healing properties [5], *C. medica* is a rich source of bioactive compounds capable of preventing and treating various diseases.

**Figure 3:**
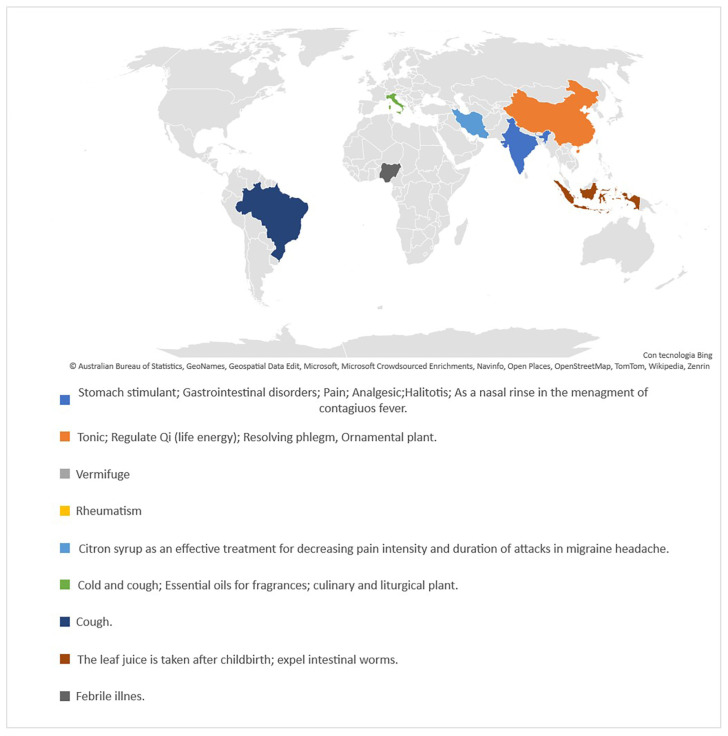
Schematic representation of traditional uses in medicine of *C. medica* in different countries.

The species is widely used in Ayurvedic medicine for antioxidant, carminative, antibacterial, anticancer, and antiviral purposes, among others [6,7]. Recently Haridas et al. [8] suggested that the herbal formulation of *C. medica* and *Zingiber officinalis* Roscoe may have good potential for reducing the viral load of SARS-CoV-2 in the nasal passages. Additionally, citron oil is widely used in Persian folk medicine for musculoskeletal, gastrointestinal, and nervous ailments [9]. Furthermore, a juice-extract syrup also showed good activity against migraines [10]. Figure 3 represents the traditional uses in medicine of *C. medica* in different countries [11,12]. 

**Figure 4:**
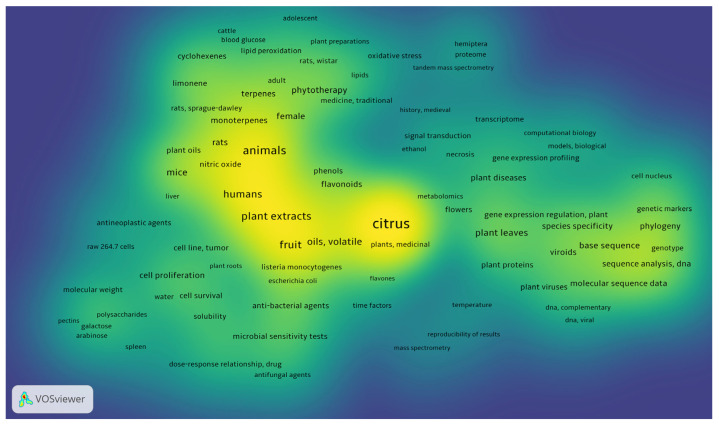
Density visualization of the main keywords in the articles analyzed.

Due to the potential role of this plant in drug discovery, this systematic review presents a careful analysis of the studies regarding *C. medica*, with a particular focus on its chemical properties and biological activity. The density visualization (Figure 4) created with VOSviewer software, version 1.6.17 (© 2022, Centre for Science and Technology Studies, Leiden University, Leiden, The Netherlands) for Windows, is proposed to offer a quick visualization of the items that concern this systematic review. The image shows the density of the keywords that appear at least twice in the selected items.

## 2. Materials and Methods

### 2.1. Search Strategy 

Based on Preferred Reporting Items for Systematic Reviews and Meta-Analyses (PRISMA) guidelines, research analysis was performed from 1 July 2022 to 31 March 2023. The search was conducted using PubMed (http://www.ncbi.nlm.nih.gov/pubmed, accessed from 1 July 2022 to 31 March 2023) and Scopus (http://www.scopus.com, accessed from 1 July 2022 to 31 March 2023), using different keywords, including “*Citrus medica*” and other terms, as follows: “carotenoids”, “flavonoids”, “coumarins”, “terpenes”, “EO”, “polysaccharides”, “antioxidant activity”, “antimicrobial activity”, “antibacterial activity”, “anti-inflammatory activity”, “hyperglycaemic activity”, “hypoglycaemic activity”, “hypocholesterolemic activity”, “hypolipidemic activity”, “cytotoxic activity”, “analgesic activity”, “anticancer activity”, “antitumoral activity”, “anticholinesterase”. The research was confined to full-text and English publications only.

### 2.2. Study Selection

The study selection included English articles containing “*Citrus medica*” in the title or abstract accompanied by keywords. Articles that treated *Citrus medica* as *Citrus bergamia* were not included in this systematic review because they are two different plant species. The exclusion criteria were as follows: review articles, articles in languages other than English, book chapters, letters, conference papers, notes, manuscripts without full text available, short reports, and short surveys. Two investigators (V.C. and N.B.) screened the literature by analyzing titles, abstracts, and full texts. In case of disagreement, another reviewer was consulted (L.M.).

### 2.3. Data Extraction

All included articles were closely examined and information related to *Citrus medica* L.’s active metabolite extraction, phytochemical profile, and biological activity was extracted. For the biological activity, in vitro cell-free and cell-based experimentation was considered. 

### 2.4. Methodological Quality Assessment

The methodological quality and the risk-of-bias assessment were carried out using a checklist adapted from Cochrane Handbook for Systematic Review of Interventions, appropriately adjusted for pre-clinical studies. Studies were analyzed based on criteria in Table 1. 

The studies that reported all the included parameters were considered of higher methodological quality. On the other hand, studies that lacked these criteria were considered at high risk of bias, while studies that did not completely fulfil the parameters were considered to have a medium risk of bias.

## 3. Results and Discussion 

### 3.1. Study Characteristics 

**Figure 5:**
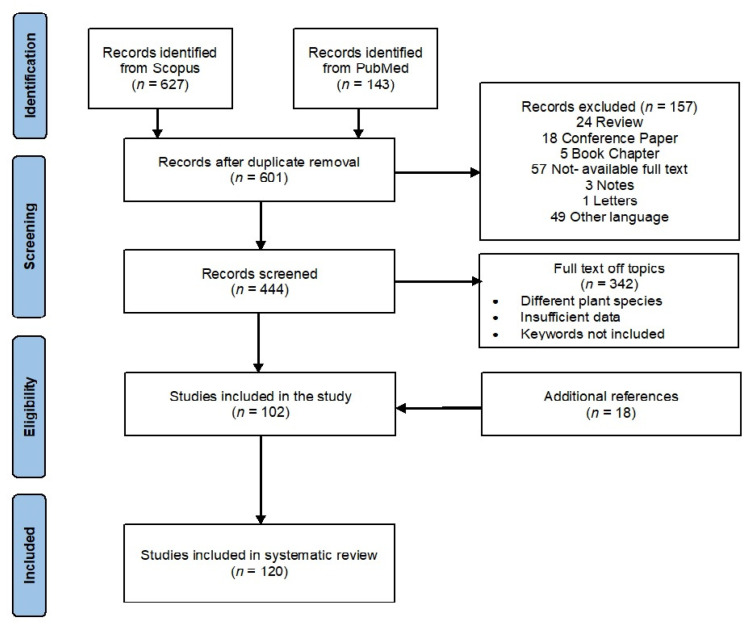
Flow diagram of the systematic review of the literature-search results based on PRISMA statement.

A preliminary survey of the literature led to the identification of 770 reports (627 from Scopus and 143 from PubMed). After checking for duplicates and articles that did not fit with the inclusion criteria, 499 results were removed, with 102 articles remaining. To these, 18 articles found in the bibliographies were added. Hence, the final reference list comprised 120 items (Figure 5).

**Figure 6:**
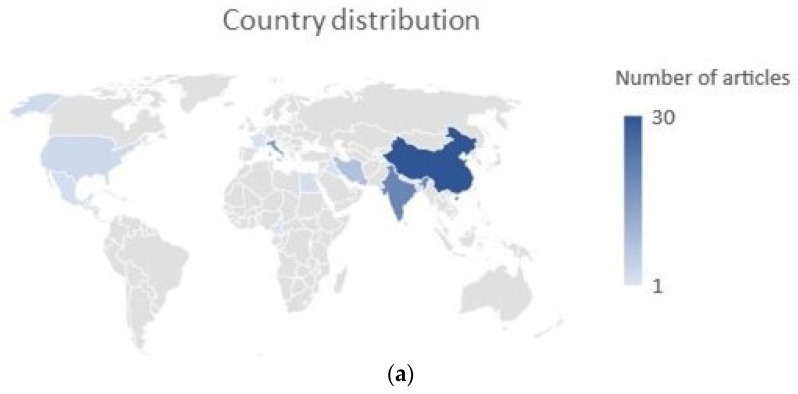

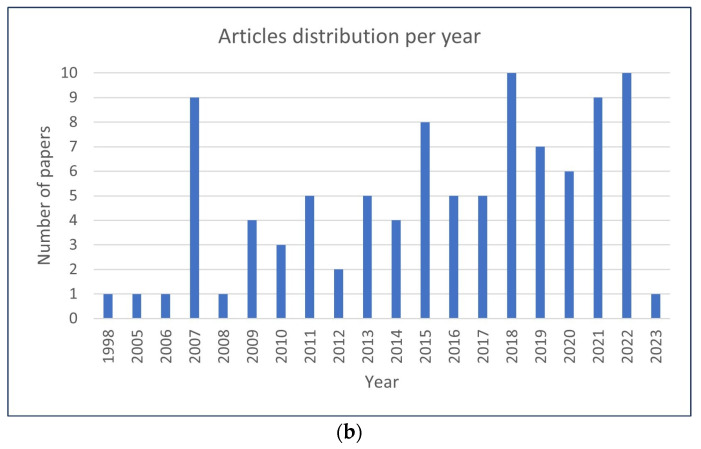
(**a**) Representation of distribution of authors’ countries of origin; (**b**) distribution of the selected studies by year of publication.

The selected papers originated in 17 countries; the country in which the greatest number of articles was published was China, followed by India and Italy (Figure 6a).

### 3.2. Phytochemistry 

**Figure 7:**
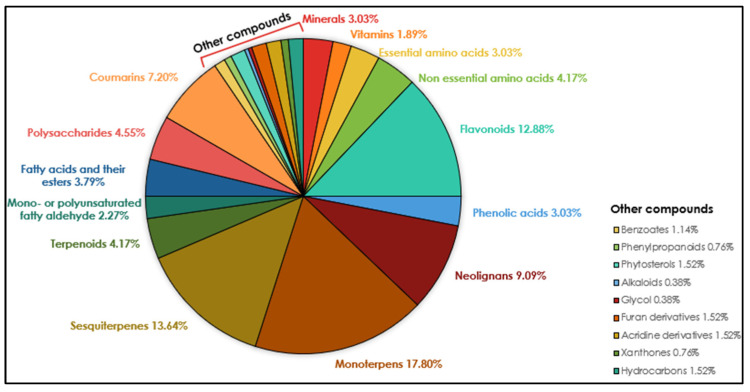
Metabolic profile of *C. medica* according to classes of compounds with percentage ranges in the plant. Values < 1.8% are grouped in other compounds.

The phytochemicals identified in *C. medica* can be classified into nutrient compounds, such as vitamins, essential amino acids, non-essential amino acids, and minerals, and non-nutritive compounds, such as flavonoids, alkaloids, terpenes, and coumarins. The diagram (Figure 7) shows the metabolic profile of *C. medica* according to the classes of compounds found in the analyzed articles. This section closely analyzes the nutritional value and chemical composition of *C. medica*, including a screening of the extractive methods used.

#### 3.2.1. Macronutrients and Micronutrients

Mahdi et al. [15] examined the nutritional composition of pulp and peel, and macronutrients, such as sugars, lipids, and proteins were determined; however, the significant contribution in terms of biological activity is due to the presence of micronutrients. The peel is richer in water-soluble vitamins than in pulp, especially in terms of vitamins B6, B1, and B2, with percentage contributions of 100 g of fresh weight (FW) to the Reference Daily Intake (C-RDI) of 779.11%, 304.69*, and 89.39%, respectively. Dadwal et al. [15] quantified the vitamin C in different parts of *C. medica* extracted by ultra-sonication and analyzed using UHPLC–QTOF–IMS with the following results: exocarp (7.95 ± 0.12 mg/100 g), mesocarp (3.05 ± 0.01 mg/100 g), endocarp (2.33 ± 0.02 mg/100 g), and seeds (3.11 ± 0.10 mg/100 g). Hasan et al. [16] analyzed the contents of vitamin C in citrus juice, finding 54 mg/100 g. Hence, it is possible to assert that juice represents the richest source of vitamin C. Furthermore, Dey et al. [3] investigated the kinetics degradation of vitamin C, indicating that temperatures above 40 °C caused the compound degradation. In addition to vitamin C, *Citrus medica* is also rich in Vitamin B, minerals (mainly present in the fruit peel and pulp), and non-essential amino acids. Table 2 reports all the nutrients found in *C. medica.*

#### 3.2.2. Polyphenols, Flavonoids, and Phenolic Acids

Flavonoids are a group of specialized metabolites with considerable health benefits, such as antiviral, antioxidant, antimicrobial, hypoglycaemic, and anti-inflammatory properties [10,23,24]. Malleshappa et al. [24] assessed the anti-inflammatory and nociceptive activity in ethanolic extract peels of some citrus fruits attributable to the high content of phenolic compounds. The flavonoids and polyphenols identified in *C. medica* can be classified into different structural categories: flavanones (naringin, narirutin, hesperidin, etc.), flavones (limocitrol 3-*alpha*-L-arabinopyranosyl-(1->3)-galactoside, scutellarein 4′-methyl ether 7-glucoside, vitexin, diosmin, etc.), polymethoxyflavones (nobiletin, tangeretin, 5-demethylnobiletin, etc.), anthocyanins (cyanidin 3-glucoside, cyanidin 3-(6′′-malonyl) glucoside, and peonidin 3-(6′′-malonyl) glucoside), flavonols (quercetin, rutin, and kaempferol, etc.), and phenolic acids, such as caffeic acid, chlorogenic acid, salicylic acid, gallic acid, benzoic acid, *trans*-cinnamic acid, *p*-coumaric acid, and *trans*-ferulic acid. These compounds are present in different percentages in all parts of *C. medica*, such as the fruits, flowers, leaves, roots, and stem barks. Dadwal et al. [15], after drying all the fruit parts and treating them with a hydroethanolic medium using UAE, detected flavonoids and other phenolic chemicals, using UHPLC–QTOF–MS, in the following order: exocarp > mesocarp > endocarp > seeds. Hesperidin was dominant in all the parts, with the highest concentration of 3307.25 mg/100 g in the exocarp extract, while naringin (295.15 mg/100 g), nobiletin (94.32 mg/100 g), and tangeretin (164.88 mg/100 g) were found in highest concentrations in the exocarp. This quantification was in agreement with the results presented by Adham [25], who demonstrated, through qualitative–quantitative analyses, that hesperidin is the dominant specialized metabolite in *C. medica* flavedo. Furthermore, a comparative study of flavedo extracts was performed by Taghvaeefard et al. [26], on two Iranian citron fruits: *C. medica cv.* macrocarpa (large citron) and *cv. medica* (small citron). The hesperidin content was 2.77 mg/g of dry weight of the fruit peel compared to 1.86 mg/g of dry weight of the flavedo from the small citron. In summary, the contents of flavone and flavonol in the small citron were twice those in the large citron obtained by macerating 200 mg of dried flavedo in methanol/acetic acid (85:15). The phytochemical profile does not depend only on the part of the plant analyzed, but also on the stage of maturation of the fruits. As reported by Menichini et al. [27], immature fruits showed a higher flavonoid contents than mature fruits. In addition to the aforementioned anti-inflammatory activity, *C. medica*’s antioxidant activity seems to be related to the amount of phenolic compounds. Specifically, a hydroalcoholic extract of *C. medica cv* Diamante demonstrated interesting antioxidant properties, probably due to the presence of high levels of hesperidin (224.3 ± 3.2 mg/kg of FW), hesperetin (203.8 ± 3.1 mg/kg of FW), rutin (156.5 ± 3.3 mg/kg of FW), quercetin (580.8 ± 3.1 mg/kg of FW), diosmin (372.53 ± 6.4 mg/kg of FW), and apigenin (941.0 ± 8.0 mg/kg of FW) [27]. The activity of these compounds has led to numerous studies on extraction from industrial by-products such as peel, seeds, and bagasse [28]. As a part of the recovery of industrial waste, the contents of flavonoids in citron seeds and their germinated shoots were compared: neohesperetin, didymin, naringenin, and hesperetin were significantly increased in the shoots after germination, with values of 14.63, 12.24, 10.51, and 20.01 mg/g DW, respectively, while the naringin and didymin were decreased compared to the citron seeds before germination [29]. Recent innovative procedures, such as microwave-assisted extraction, supercritical carbon dioxide, enzyme-assisted extraction, pulsed electric field, sub-critical water extraction, and solar-energy-assisted extraction have been proven to be good methods for the up-scaled application of the recovery of bioactive components present in low concentrations [30]. In this vein, Govindarajan et al. [31] investigated the optimum condition using a response surface methodology on the pectin yield from dried *C. medica* peel with the following parameters: microwave power of 480 W, irradiation time of 20 s, and dilution factor of 1:10 weight/volume (*w*/*v*). Recently, six new neolignans were identified and characterized by Ma et al. [32], compared to common extractions; in this case, the fruits (9.5 kg) of *C. medica* var. *Sarcodactylis* were air-dried, smashed, and extracted with 95% EtOH heating under reflux at 110 °C for 4 h with an electric heating jacket. The chemical properties of the polyphenols, flavonoids, and phenolic acids are reported in Table 3.

#### 3.2.3. Terpenes

Terpenes are a class of natural products formed by different isoprene units (C_5_H_8_) that determine structural classifications in monoterpenes, diterpenes, sesquiterpenes, triterpenes, and tetraterpenes. The EO, obtained primarily from the flavedo of *C. medica*, is rich in these specialized metabolites. In recent years, the attention focused on these molecules used as perfumes and for the preservation of foods has considerably increased, thanks to their antimicrobial activity against *Saccharomyces cerevisiae* [44]. Additionally, other studies have evaluated the anti-inflammatory and antioxidant activity associated with these molecules [28]. The composition of EO depends on several factors, such as the extraction method, different stages of fruit maturity [45], environmental factors, geographical location, and genetic variations. All these variables make the comparison between studies complex [46]. In fact, the peel oil of the *C. medica* var. *Sarcodactylis* profile reported by Jing et al., including limonene (41.8%), geranial (17.9%), neral (13.6%), citronellal (4.4%), and nerol (4.1%), was different from that reported by Venturini et al., using *C. medica* cv. *Corsican* [47]. However, in all these studies, limonene and *γ*-terpinene were the most abundant compounds identified in *C. medica* EO. Their quantity depends on the maturity stage of the fruit. In particular, the highest concentration of limonene (36.37%) was found in the immature stage, while the highest content of *γ*-terpinene (25.23%) was reported in the intermediated stage, but was reduced in mature fruits.

Furthermore, in the mature stage, the monoterpene-hydrocarbon content increased, but the amount of sesquiterpene hydrocarbons and total sesquiterpenes decreased [45]. According to Taghvaeefard et al., the main constituent of EO from the flavedo in *C. medica* var. macrocarpa was limonene (89.39%), while in *C. medica* var. *medica*, limonene (48.59%), linalool (22.98%), and linalyl acetate (8.21%) were the main components detected [26]. Regarding the extraction condition, a low yield represents a limiting factor for the recovery of EOs. Poiana et al. performed three different extraction techniques on *C. medica* cv. Diamante: the commonly used hydro-distillation of fresh and dried peel, supercritical carbon dioxide extraction (SCF–CO2), and solvent extraction using pentane. The contents of monoterpenes and limonene were higher with the hydro-distillation but decreased with the SCF–CO2, with which it was instead possible to observe an increase in sesquiterpenes. The reason for this is that mainly volatile molecules were extracted in the hydro distillates, while the high density of SCF–CO2 increased the solubility of the non-volatile compounds [48]. According to Bartolo et al., the best extraction method is the abrasion of rinds, except for limonene, which has a better yield with manual squeezing [49]. These techniques allow the extraction of a quantity of active metabolites that is greater than that of the oil obtained by simple maceration with hexane, which, as reported by Conforti et al., led to the identification of 45 compounds and an extraction yield of 0.13% [50]. In addition, Xing et al. obtained an extraction yield of 0.48% by using ultrasound-assisted hydro-distillation (UAHD) [51], while Wei et al. [52] demonstrated that under the optimal extraction parameters (microwave irradiation power, microwave irradiation time, and homogenization time) the essential oil yield (1.65% ± 0.05%) from solvent-free microwave extraction was 27.91% higher than that from hydro-distillation (HD) (1.29% ± 0.03%), which was probably due to the special heating mode of the microwave. Several studies also reported a comparison of yields obtained with hydro-distillation vs. steam HD. Jing et al. showed a low yield of oil obtained by the steam distillation of citrus peel (0.64 ± 0.07 g of oil/g) [47]. Wu et al. compared different fruit stages of *C. medica*, demonstrating that the EO-extraction yield ranged from 2.39 ± 0.08% *w*/*w* in the immature stage to 3.57 ± 0.12% *w*/*w* in the mature stage, which was higher than that reported by Peng et al. [53] (0.45%). The reasons for this difference could be the geographical origin and the different methods of extraction; in fact, in the first case, the EO was obtained by HD from fresh fruits grown in China, while in the second study, the oil was obtained by the steam-based hydro-distillation of dried fruits cultivated in Japan. Vitalini et al. [54] described different exocarp EO and hydrolate (HY) compositions. The volatile profile of the EO was characterized by limonene (66.9%) and *γ*-terpinene (20.0%) as the most abundant compounds, while *α*-terpineol (44.7%), and terpinen-4-ol (21.6%) were found in the HY extract. Furthermore, several minor monoterpene components, such as *α*-thujene (0.2%), *α*-pinene (0.6%), *β*-thujene (0.1%), *β*-myrcene (0.9%), *β*-pinene (0.8%), (+)-4-carene (0.2%), R-(+)-citronellal (0.1%), nerol acetate (0.2%), and geranyl acetate (0.1%), which were found in the EO, were missing in the hydrolate (HY). Instead, other compounds, such as *β*-terpinene (1.0%), linalol (5.7%), thymol (1.9%), and piperitenone (0.4%), were detected only in the HY. Furthermore, as with many other citrus fruits, *C. medica* is an important source of carotenoids, also called tetraterpenoids, which are made up of a carbon skeleton characterized by six isoprene units. Fanciullino et al. [55] analyzed the carotenoid contents of twenty-five citrus varieties and reported that in *C. medica*, *β*-cryptoxanthin was found without cis-violaxanthin, while in Citrus maxima, only cis-violaxanthin was found, with a lack of *β*-cryptoxanthin. The total carotenoid content in the extracts of Etrog citron and Diamante citron juices was identified by a comparison of their retention times and UV/vis spectra, showing 0.227 mg lycopene/L and 0.019 mg lycopene/L respectively. Other typical specialized compounds abundant in citrus fruits and the Rutaceae family are limonoids, which chemically constituted by variations in the structure of the furanolactone core. The most frequently present components in *C. medica* are limonin and nomilin, which are responsible for the bitter taste of the genus Citrus. Lim et al. [56] investigated the optimum conditions for the enzymatic hydrolysis of citron waste juice using the response surface methodology: the highest contents of limonin and nomilin were 3.49 mg/100 g (extraction conditions: pH 4.51, temperature 50.30 °C, time 48.34 min, and 0.21% yield) and 1.56 mg/100 g (extraction conditions: pH 4.59, temperature 50.08 °C, time 66.07 min, and 0.30%), respectively. The terpenes identified in *C. medica* are shown in Table 4. They are grouped into their respective categories based on the number of isoprene units: monterpenes, diterpenes, triterpenes, tetraterpenes, and polyterpenes.

#### 3.2.4. Coumarins

Coumarins are natural phytochemicals that are widely distributed in plants and are strongly related to numerous pharmacological activities. They belong to the lactone family, consist of a benzene ring fused to a *α*-pyrone ring, and can be classified into different subtypes. Several phytochemicals studies have shown the abundance of coumarins in citrus fruits; 5,7-dimethoxycumarin was found to be the most abundant coumarin (876.7 ± 4.7 µg/g) in a fruit extract (var. *Sarcodactylis*) using pressurized liquid extraction with methanol at 90 °C. This represents, together with hesperidin, a marker for the quality control of *Citrus* fruits [40]. Vitalini et al. also identified 5,7-dimethoxycumarin as the most abundant compound (50.6%), followed by 2-pyrone (23.4%), in a methanolic extract of the exocarp of a variety of *C. medica* from Switzerland. Furan derivatives were the main class of compounds detected by GC–MS, of which 5-hydroxymethylfurfural was the main exponent, with relative percentages of 14.7% and 24.8% in the exocarp extract and in the mesocarp extract, respectively. In addition, 2-Furanmethanol (3.9% for the exocarp extract; 6.7% for the mesocarp extract) and furaneol (3.1% for the exocarp extract; 3.6% for the mesocarp extract) were present in both extracts. Furthermore, 2-pyrone (33.1%) and 2,3-butanediol (23.7%) were the main component non-furanoic derivatives present in the mesocarp [54]. Coumarins were also detected in the root bark of *C. medica*. Wang et al. identified two coumarins, xanthyletin and xanthoxyletin, by micellar electrokinetic capillary chromatography; their amounts (1.6 mg/g and 0.7 mg/g, respectively) were lower than those found in other *Citrus* species, such as *C. reticulata* (3.6 mg/g and 1.5 mg/g, respectively) [60]. In *Citrus* fruits, other coumarins have also been identified in lower amounts or in traces, such as 7-hydroxycoumarin, 6,7-dimethoxycoumarin, and bergapten [40]. All the other coumarins found in *C. medica* are listed in Table 5.

### 3.3. Other Compounds

Other compounds were identified in C. medica, and they are classified in the following Table 6. In addition, several authors isolated and characterized new polysaccharides (listed in Table 7), which may be endowed with potential bioactivities.

### 3.4. Biological Activity

After establishing the large presence of bioactive compounds, it is necessary to investigate the capacities of specific compounds or extracts obtained by different extraction methods and different parts of *C. medica* to achieve a defined biological effect. Antioxidant and antimicrobial activities have been widely studied, particularly analgesic, anti-inflammatory, and hypoglycemic activities. All these activities are reported in the Table 8.

#### 3.4.1. Antioxidant Activity

Antioxidants are compounds that are able to neutralize free radicals, which can damage the body’s cells [93]. The *C. medica* has significant antioxidant properties due to the presence of an excellent antioxidant, ascorbic acid, in addition to known phenolic compounds, flavonoids, carotenoids, and heteropolysaccharides, which contribute to antioxidant capacity in citrus fruits. Several studies have evaluated the antioxidant activities of different parts of *C. medica* in combination with the total contents of phenols and flavonoids, widely known as molecules with marked antioxidant activity [36,94,95,96]. In fact, Luo et al. [41] investigated the major flavonoids of finger citron, prepared by continuous phase-transition extraction (CPE), purified with AB-8 macroporous resins and then identified by UHPLC–QTOF–MS, showing good radical-scavenging activity on an in vitro test. The scavenging capacities of DPPH and ABTS were investigated from 0.1 mg/mL to 1 mg/mL using ascorbic acid as the standard. At the concentration of 1.0 mg/mL, the DPPH radical scavenging was 90.24%, and no significant differences were found from that of 0.8 mg/mL, which was 89.86%, compared with the 94.74% inhibition of ascorbic acid. At a low concentration (0.2 mg/mL), the purified extract showed a scavenging capacity of ABTS radicals equal to 87.94% compared with the ascorbic acid. Considering the wide use of the fruit in the diet, the result demonstrated with the ORAC (oxygen radical absorbance capacity) test, of 928.64 μmol TE (Trolox equivalent)/g, is of significant interest. Furthermore, Mondal et al. [42] reported a radical-scavenging potency of *C. medica* fruit methanol extract (IC_50_ 112.18 μg/mL) using ascorbic acid as the standard (IC_50_ 25.53 μg/mL). On the other hand, the IC_50_ values calculated for the *C. medica*-fruit methanol extract and ascorbic acid for the nitric oxide (NO) radical-scavenging assay were 117.38 μg/mL and 45.23 μg/mL, respectively. As reported by Mahdi et al. [15], Foshou (*C. medica* var. *Sarcodactylis*) peel showed significantly higher antioxidant properties and nutritional contents than the pulp. The aqueous extracts of Foshou peel and pulp contained 227.45 mg GAE (gallic acid equivalent)/100 g FW and 88.76 mg GAE/100 g FW of total phenolic compounds, with DPPH scavenging-activity levels of IC_50_ 22.79 μg GAE/mL and 54.74 μg GAE/mL, respectively. The antioxidant capacities of the Foshou peel and pulp was 214.81 mg TE/100 g FW and 71.53 mg TE/100 g FW, respectively. Pallavi et al. [97] also compared the antioxidant activities of peel and pulp extracts from five varieties of *C. medica*: citron, sour orange, lemon, pomelo, and orange. The citron peel and pulp demonstrated the highest phenolic contents (66.36 μg GAE/mg for the peel and 51.21 μg GAE/mg for the pulp) and flavonoid contents (40.17 μg catechol/mg for the peel and 37.9 μg catechol/mg for the pulp) compared to the other varieties. Similar results were obtained for the total antioxidant content (140.17 µg ascorbic acid equivalent/mg for the peel and 116.11 µg ascorbic acid equivalent/mg for pulp) and the DPPH peel and pulp radical scavenging (EC_50_ (half maximal effective concentration) 827.26 µg/mL and 4089.64 µg/mL, respectively); however, the orange pulp was the least active (EC_50_ 3628.44 µg/mL), followed by the citron pulp (EC_50_ 4089.64 µg/mL).

The antioxidant activity of peel was extensively studied. As reported by Menichini et al. [33], hydroalcoholic peel extract inhibited both DPPH and ABTS radicals with IC_50_ values of 0.80 ± 0.07 and 3.48 ± 1.0 mg/mL, respectively, compared with ascorbic acid (0.002 ± 0.01 and 0.009 ± 0.0003). In the *β*-carotene-bleaching test, the peel extract reduced the *β*-carotene discoloration, exhibiting good activity (IC_50_ 0.23 ± 0.002 mg/mL) with propyl gallate, with IC_50_ 0.001 ± 0.0001 mg/mL. Conforti et al. also reported the activity of a peel *n*-hexane extract, showing an IC_50_ of 147 ± 1.23 µg/mL against DPPH radicals with ascorbic acid as the positive control (2.00 ± 0.03 IC_50_ µg/mL) and an antioxidant activity of IC_50_ 3.00 ± 0.05 µg/mL, as evaluated by a *β*-carotene bleaching assay at 30 min, with propyl gallate as positive control (IC_50_ 1 ± 0.04 µg/mL). The authors reported different results, probably due to the different extraction used. 

The antioxidant activities of juice, leaves, and flowers has not been extensively studied but is equally important. Dey et al. analyzed the antioxidant power of 0.1 mL of concentrated juice from three cultivars, Diamante, Balady, and Corsican, with the following test data: 72.00 ± 0.82% for DPPH radical-scavenging activity and 309.08 ± 3.06 mg GAE/g for TPC [3]. Furthermore, the methanol-extract leaves showed antioxidant activity (EC_50_ 102.9 µg/mL), when compared to the ascorbic acid (EC_50_ 49.28 µg/mL) [38].

The antioxidant capacity depends on the species, cultivar, stage of maturation, pedoclimatic conditions, and other agronomic factors. Wu et al. [45] reported the antioxidant activity of EO obtained from *C. medica* fruits in different maturation stages. A stronger DPPH radical inhibition was reported for the EO of the fruit in the immature stage, of 78.4 ± 2.6%, compared to that in the mature stage, of 63.8 ± 2.1%. Furthermore, the EO obtained from the immature stage showed greater reducing power by converting the Fe^3+^/ferricyanide complex into the ferrous form than those collected during the mature stage. The differences between these results may have been due to the different chemical compositions, since even if the activity is principally attributed to the most abundant compounds, the antagonistic effect of a compound present in smaller amounts must be considered. Such results can also be affected by changes in humidity and temperature during different seasons. To support this, Menichini et al. [27] demonstrated that the highest total contents of phenols and flavonoids were in immature fruits, although the greatest content was found in flowers (398.0 ± 3.2 and 266.9 ± 7.2 mg/100 g, respectively), followed by leaves (401.6 ± 5.1 and 97.5 ± 2.8 mg/100 g respectively). Despite the greater content present in the flowers, the best DPPH scavenging activity was demonstrated by the mesocarp of immature fruits (IC_50_ of 382.0 µg/mL), followed by flower and leaf extracts, with IC_50_ values of 425.0 and 502.0 µg/mL, respectively. By contrast, the flowers showed the highest inhibition of linoleic acid oxidation (IC_50_ value of 2.8 µg/mL) after 30 min of incubation. Indeed, Taghvaeefard et al. [26] compared the antioxidant activities of flavedos from two Iranian citron fruits, proving that the antioxidant activity was higher in the large citron, *cv.* macrocarpa (IC_50_ 170.142.5 mL/L), than in the small citron, *cv. medica* (IC_50_ 280.125 mL/L). Although the total contents of phenols were comparable, and the content of flavonoids in the small citron was twice that in the large citron, this confirmed the different compound contents in the different *Citrus* varieties and the synergistic activities of some compounds. These differences could be attributed to the different chemical compositions of the same *Citrus* species in different regions. Many studies have also investigated the antioxidant activity of EO. Vitalini et al. reported good radical-scavenging activity against the ABTS of EO and methanol exocarp and mesocarp extracts of 54.1 ± 0.2%, 55.8 ± 5.4%, and 52.0 ± 0.4%, respectively. The methanolic exocarp and mesocarp extracts also showed good activity towards the stable DPPH radical, reporting a percentage of inhibition of 55.7 ± 1.20% for the exocarp extract and of 46.7 ± 0.82% for that of the mesocarp. These activities can be associated with the presence of compounds such as flavonoids and polyphenols. In fact, the authors reported a total exocarp-extract polyphenol content of 2.52 ± 0.07 mg GAE/g in the fruit part compared to 1.74 ± 0.02 mg GAE/g in the fruit part of the mesocarp, along with a total flavonoid content of 2.20 ± 0.26 mg QE/g in the fruit part of the exocarp versus 1.50 ± 0.06 mg QE/g in the fruit part of the mesocarp extract [54].

On the other hand, the highest antioxidant activity of the EO from *C. medica* var. macrocarpa Risso was measured by Ghani et al. at the over-ripe stage (76.08% ± 0.51% radical-scavenging activity), which was probably due to specific compounds of a different variety [98]. In agreement with Guo et al., the EO of *Citrus medica cv.* Sarcodactylis showed the highest antioxidant activities, of 77.2%, in a DPPH assay [47].

Overall, antioxidant bioactive compounds are often confirmed by in vitro assays. However, they are characterized by poor absorption through biological barriers. To enhance their beneficial effects and to overcome this limitation, Zhao et al. [35] developed Ca–alginate microbeads with the polysaccharide-filler-controlled delivery of phenolic compounds. A *C. medica*-fruit extract was analyzed for the total phenolic (31.60 ± 0.35 mg GAE/g) and flavonoid (15.38 ± 0.02 mg RE (rutin equivalent)/g) contents. In addition, phenolic compounds entrapped in microbeads were identified using UHPLC–DAD–QTOF–IMS after in vitro digestion. The quantification results showed that the alginate extract and pectin filler (APE) had the highest amounts of phenolics, particularly hesperidin (264.11 mg/100 g), tangeretin (29.67 mg/100 g), and nobiletin (127.14 mg/100 g) [99]. Recently, Peng et al. [61] extracted a crude polysaccharide from the residues of *C. medica* var. *Sarcodactylis* (CMSPB80-1). The CMSPB80-1 exhibited the highest DPPH radical-scavenging rate, of 47.45% (3.2 mg/mL), while the highest ABTS radical-scavenging rate was 49.58% (3.2 mg/mL), within a concentration range of stable free radicals from 0.05 to 3.2 mg/mL. Similarly, Luo et al. [66] isolated from fresh fruits of *C. medica* var. *Sarcodactyilis* a new type of galactorhamnan, named K-CMLP (*Citrus medica* polysaccharide), consisting of rhamnose, galactose, and glucose, which exhibited free-radical scavenging (IC_50_ 2.5520 mg/mL) that was lower than those other extracts obtained from whole fruit, according to a DPPH test,. Previously, Wu et al. [67], showed an increase in O_2_^•−^ scavenging ability in citron-fruit polysaccharides, from 7.7% to 73.5%, when the concentration increased from 0.05 mg/mL to 0.80 mg/mL. Additionally, Wu et al. [100] studied three drying methods, freeze drying, hot-air drying, and vacuum drying, to enhance the physicochemical and antioxidant properties of finger-citron polysaccharides. The results showed that the maximum yield (88.7% radical-scavenging activity) was obtained by freeze-drying, suggesting that it may be possible to obtain a novel polysaccharide with strong radical-scavenging capacity through the optimization of freeze-drying parameters. These studies demonstrate that antioxidant activity is linked not only to phenolic compounds and their derivatives, but also to heteropolysaccharide molecules, and represents a reuse of waste resulting from fruit processing. On this basis et al. [68] investigated the total phenolic content of *Citrus-aurantium*-L.-, *C.*-*limon*-, and *C.-medica*-by-product extracts, reporting values of 92.0 ± 4.8, 41.7 ± 13.1, and 25.8 ± 2.8 mg GAE/g of DW, respectively. They also evaluated the total flavonoid contents (TFCs) of citrus-by-product extracts, reporting the highest content for *C. aurantium* (161.09 ± 0.2 mg QE (quercetin equivalent)/g), followed by *C. limon* and *C. medica* (63.97 ± 0.3 and 29.29 ± 5.6 mg QE/g, respectively). In addition, the antioxidant capacities of citrus by-product extracts were investigated: the highest DPPH radical-scavenging activity was detected for the by-product of the extract of *C. aurantium* (90.1 ± 0.6%), followed by those of the extracts of *C. limon* (44.6 ± 1.2%) and *C. medica* (43.8 ± 0.3%), according to the total-phenolic- and flavonoid-content results.

The ability of *C. medica* to act on intracellular ROS concentrations was investigated in human epidermal keratinocytes (HaCaT) stimulated by H_2_O_2_. The extract obtained from the whole fruit significantly decreased the intracellular ROS compared to the positive control, probably acting on the endogenous antioxidant defenses. In fact, as shown by the authors, the superoxide dismutase (SOD) and catalase (CAT) enzymes were upregulated by the extract [101]. 

#### 3.4.2. Antibacterial, Antiviral, and Antifungal Activity

Bacteria may develop resistance to conventional drugs; therefore, there is an increasing need to find new antimicrobial agents. Medicinal plants are rich sources of bioactive compounds which also display antimicrobial activities [102]. Among the many biological properties of *C. medica*, its antimicrobial properties are undoubtedly the most heavily investigated, probably due to the strong presence of phytochemicals, such as citral, linalool, and limonene [103,104,105], which are endowed with antimicrobial effects. Most of the relevant studies concern citron’s antibacterial activities, but some researchers have also focused their attention on the antiviral properties of this species. For example, *C. medica* var. Sarcodactylis EO was screened for its antiviral activity by El Hawary et al. [69]. The investigation was carried out by incubating the Madin Darby canine kidney (MDCK) cell line with Avian influenza A virus (H5N1). At a concentration of 0.5 µg/µL, the EO from leaves showed 95% inhibition, while the inhibition by the fruit EO was lower (50%). According to previous studies on the antiviral activity against H5N1, limonene, which is present in high quantities in the citron EO, seems to be one of the main factors responsible for this activity [104]. Many researchers investigated the ability of *C. medica* EO to reduce food spoilage. Belletti et al. [44] investigated the effect of citron EO, citral, and (E)-2-hexenal on the spoilage of noncarbonated beverages inoculated with different amounts of a *S. cerevisiae* strain combined with a mild heat treatment (55 °C). They found that the citron EO had the highest capacity to prevent yeast growth in terms of time, which was probably due to the presence of other molecules with synergistic actions, such as *β*-pinene and limonene. The authors investigated the antimicrobial potential of *Salmonella enteritidis*, *Escherichia coli*, and *Listeria monocytogenes* in fruit-based salads packaged in plastic containers. Regarding noncarbonated beverages, the citron EO showed the best results, avoiding the undesirable cytotoxicity of the citral, since the shelf life of the fruit-based salads was doubled [70]. Wu et al. [71] demonstrated, in a disc-diffusion test, that the fungal-inhibitory activity of EO depends on the contents of the compounds. Compared to the flower and fruit EOs, the leaf EO is richer in oxygenated monoterpenes, to which the antifungal activity can be attributed. The former (inhibition zone ranging from 8.54 ± 1.27 mm to 15.26 ± 2.16 mm) showed a smaller inhibition zone than the latter (inhibition zone > 90.00 mm) against mold growth on Chinese steamed bread. The effect of the EO obtained from *C. medica* fresh finger fruits on lactic acid bacteria isolated from vacuum-packed cooked and cured sausages was assessed by Khorsandi et al. The results were encouraging because the EO was able to act against the bacteria by reducing the spoilage of the food [72]. The effects of finger-citron EO on food-borne bacteria (*E. coli*, *St. aureus*, *Bacillus subtilis*, and *Micrococcus luteus*) were investigated by Li et al. [73]. They demonstrated a stronger effect of the extract on Gram-positive (minimum inhibitory concentrations (MIC) ranging from 0.625 to 1.25 mg/mL) than on Gram-negative bacteria (MIC 2.5 mg/mL). The *C. medica* EO can also be applied in the wine industry; Mitropoulou et al. [74] demonstrated that the spoilage and microbial growth of wine (inoculated with *Gluconobacter cerinus*, *Oenococcus oeni*, *Pediococcus pentosaceus*, *Dekkera bruxellensis*, *Candida zemplinina*, *Hanseniaspora uvarum*, *Pichia guilliermondii*, or *Zygosaccharomyces bailii*) was considerably delayed after treatment with EO (18 days, compared to the 9 days used for the control). The MIC of the *C. medica* EO on *Panax notoginseng* Burkill root fungi (*Fusarium oxysporum*, *Fusarium solani*, and *Cylindrocarpon destructans*) were 9.38 mg/mL, 12.05 mg/mL, and 8.44 mg/mL, respectively; these values were not significantly higher than those of the positive control, hymexazol (MIC values 0.12 mg/mL, 0.16 mg/mL and 0.18 mg/mL) [75]. The EO from the aerial parts of *C. medica* was not effective on *Yersinia enterocolitica* O9, *Proteus* spp., *Klebsiella pneumoniae*, or *E. coli*, as demonstrated by Al-mariri and Safi [76]. Mitropoulou et al. [77] analyzed the EO phytochemical composition, and limonene was the main constituent identified by SPME GC/MS (88%) and GC/MS (64%). The antimicrobial effects of limonene and those of the whole phytocomplex on several bacteria and fungi were compared: the MIC values were significantly (*p* < 0.05) lower for the *C. medica* EO (<2000 mg/L) than for the limonene (>5000 mg/L), probably due to the synergistic effects of other components. The EO from *C. medica* var. *Sarcodactylis* Swingle, cultivated in China, showed strong antimicrobial activities against the bacteria and fungi tested (MIC ranging from 1 and 4% *v/v*), probably due to the strong presence of linalool, which showed lower MIC (0.125–0.5% *v/v*) compared to the other compounds identified [46]. Zang et al. [78] compared the effect of the extraction technique on the antimicrobial activity of the EO from *C. medica* var. *Sarcodactyilis*. They found an increased inhibition of biofilm formation from *S. aureus* in the ultrasonic/microwave-assisted hydro-distillation and hydro-distillation extraction (100% of inhibition at 0.75 mg/mL) compared to the solvent extraction (83% at the same concentration). This difference was attributed by the authors to the high presence of volatile components in the hydrodistilled extracts.

Three kinds of fingered citron (*Citrus medica* L. var. Sarcodactylis Swingle) EO showed different antimicrobial activities: the Gold, Cantonese, and Sichuan fingered citron EOs showed the strongest antibacterial activity on *S. aureus*, *E. faecalis*, and *E. coli*, respectively [79]. The Eos were nano-functionalized by inclusion in sulphur- and aluminum-oxide nanoparticles, showing the highest inhibitory-activity levels on *Salmonella typhi* (21 mm) and *F. oxysporum*, respectively. A good increase in growth inhibition was observed for EO in combination with antibiotics [80]. The nanoemulsion of the EO from *C. medica* L. var. *Sarcodactylis* was developed by Li et al., who demonstrated the increased antibacterial activity of an extract against *E. coli*, *B. sublitis*, and *S. aureus.* The same was not observed with fungi, whose growth was reduced more by the free EO than the nanoemulsion [81]. 

Citron-peel extracts obtained with different solvents (acetone, DMSO, methanol, petroleum ether) were tested for their antibacterial capabilities on several pathogenic microorganisms, which were either fungi or bacteria. Overall, the solvent that made it possible to obtain the extract with the strongest antimicrobial activity was the DMSO, since a larger inhibition zone than that of the control (Gentamycin for bacteria and Ketoconazole for fungi) was observed [105]. Ethyl acetate and ethanol 80% peel extracts exhibited larger inhibition zones (10 mm and 22 mm, respectively) at 100 mg/mL compared to juice extracts, against which the bacterium showed complete resistance [83]. A peel extract of the *C. medica* variety grown in the Kumaun region in Uttarakhand, India was tested for its antimicrobial capacity against *P. aeruginosa* without any results, while the pulp and juice extracts from the same variety were active against this bacterium. The researchers also tested root, leaf, and bark extracts on several bacterial strains. The extracts that showed the strongest activities were the root and juice extracts with, 19-nn and 17-mm inhibition zones, respectively, which even higher than that of the standard drug (chloramphenicol, 14 mm) [84]. In contrast, Sharma et al. stated that a *C. medica*-juice extract had no effect on the growth of any of the bacteria they tested (*B. subtilis*, *S. aureus*, *Es. Coli*, and *K. pneumoniae*), while it was active against fungi (*Aspergillus niger* and *C. albicans*) [85]. In 2015, Shende et al. synthetized copper nanoparticles containing the juice of *C. medica* collected in Amravati, Maharashtra, and India. They demonstrated that the encapsulation of the extract in nanoparticles strongly increased the inhibitory activity of the *C. medica* against the tested bacteria, *E. coli*, *K. pneumoniae*, *P. aeruginosa*, *Propionibacterium acnes*, and *Salmonella typhi*, and fungi, *Fusarium culmorum*, *F. oxysporum*, and *F. graminearum* [87]. Castillo et al. assessed the antimicrobial activity of *Campylobacter jejuni* from *C. medica* by-products (peel, seeds, and bagasse). The extract reduced the swarm motility (35–40%) and biofilm formation (60–75%). Quorum sensing was performed by measuring the Autoinducer-2 activity, which was reduced by about 90% by the extract [86]. The study continued with the analysis of the effects of *C. medica* by-products on *C. jejuni* adherence (which was from reduced by 50.8% to 91%) and invasion (reduced by 85.1% to 94.8%) to human tumor cells (HeLa), while the gene expression of adhesion (cadF) and invasion (ciaB) molecules was significantly (*p* ≤ 0.05) reduced [68]. Peel and pulp extracts of *C. medica* cv. *medica* and *C. medica* cv. Salò were found to exert antibacterial activities against *E. coli*, *L. monocytogenes*, *P. aeruginosa*, *S. aureus*, and *Pectobacterium carotovorum* (due to the high contents of phenolic acids and flavonoids), with a strong capacity to inhibit biofilm formation, especially on *L. monocytogenes* [34]. Keerthana et al. demonstrated that the use of nanoparticles could be a good strategy to enhance the antimicrobial activity of an extract. In fact, the inclusion of *C. medica*-peel extract in ZnO nanoparticles increased the sensitivity of the bacterial strains, with the largest inhibition zone against *S. sannanesis* (25 mm), followed by *B. subtilis* (24 mm), *Pseudomonas aeruginosa* (23 mm), and *Salmonella enterica* (22 mm) [82]. Researchers attributed the strong bactericidal capacity of ZnO nanoparticles to their production of reactive oxygen species (ROS) in water suspensions [106]. Nanomaterials were obtained from *C. medica* by Selvaraju et al. Specifically, starting with the fruit extract, they obtained carbon quantum dots (CQDs) and tested their capacity to act on the pathogen *P. aeruginosa.* As shown by the crystal violet staining assay, the CQDs inhibited the growth of the bacteria with a MIC of 1.25% (*v*/*v*) [88]. A cyclic peptide previously isolated from the fruit peel of *C. medica* var. Sarcodactylis Swingle was synthetized by Dahiya and Kumar [91]. They evaluated the antimicrobial activity of the new peptide against the Gram-positive bacteria *B. subtilis* and *Staphylococcus aureus*, as well as the Gram-negative bacteria *P. aeruginosa* and *E. coli*, in comparison to the standard drug, ciprofloxacin. The peptide was active only against *P. aeruginosa*, showing a similar MIC value to the ciprofloxacin (6 μg/mL), with an inhibition zone 28 mm in diameter, compared to the 25 mm displayed by the reference drug. In addition, the peptide was also active against *Candida albicans*, with a 22-mm-diameter inhibition zone, compared to the 20 mm displayed by the griseofulvin. Chromatographic fractionation and the enrichment of phenolic components from *C. medica* var. Sarcodactylis fruit strongly increased the antibacterial and antibiofilm capacity of the species against *S. aureus*, with a 100% inhibition of biofilm formation at 2.0 mg/mL [21]. A *C. medica* var. Sarcodactylis exocarp ethanol extract showed stronger inhibition against *Bacillus cereus* (MIC 2.5 mg/mL) than against *E. coli* (MIC 10 mg/mL). The higher content of coumarin may explain the stronger antimicrobial activity of the exocarp extract [7].

#### 3.4.3. Cytotoxic Activity

As stated by the World Health Organization, together with cardiovascular diseases, cancer is an important cause of mortality worldwide, which, according to recent trends, could even rise above heart diseases as the leading cause of death [107]. Despite the great therapeutic advances in traditional cancer therapies, they feature several disadvantages, such as systemic toxicity, drug resistance, and side effects [108]. Natural products have been found to possess antitumor and tumor-preventive properties. *Citrus* species, including *C. medica*, are traditionally used for anticancer applications [109]. 

Nair et al. used Dalton’s lymphoma ascites (DLA) cells to evaluate the toxicity of *C. medica* peel-oil and peel-water extracts. The peel-oil extract induced 30.2 ± 2.2% and 73.3 ± 2.6% cell death at 25 µg/mL and 50 µg/mL, respectively, while the *C. medica* peel-water extract was less toxic, showing 56.5 ± 3.6% inhibition at 50 µg/mL [90].

The cytotoxic effects of the EOs extracted from two different cultivars of *C. medica* (cv. ‘liscia’ and cv. ‘rugosa’) grown in the Campania region, Italy, and limonene, as the major component (67.2% for *C. medica* cv. liscia and 62.8% for *C. medica* cv. rugosa), was evaluated on a human neuroblastoma cell line (SH-SY5Y). The limonene and *C. medica* cv. ‘rugosa’ EO showed an IC_50_ > 2000 µg/mL, while the *C. medica* cv. ‘liscia’ EO showed an IC_50_ of 718.2 µg/mL [110]. An exocarp ethanol extract from *C. medica* var. Sarcodactylis was more toxic (EC_50_ 1.76 mg/mL) than a mesocarp extract (EC_50_ not attained) in a HL60 leukemia cell line [54]. This might be explained by the strong presence of coumarins, which have been reported to have good anticancer activity [111].

Dahiya and Kumar [91] synthesized a cyclic peptide, sarcodactylamide, previously isolated from the fruit peel of *C. medica* var. Sarcodactylis Swingle. The synthesis was carried out by coupling two tetrapeptide units (Boc–Leu–Pro–Trp–Leu–OMe and Boc–Ile–Ala–Ala–Gly–OMe) after the deprotection of carboxyl and amino terminals and the cyclization of the linear octapeptide segment. The authors proved that the new peptide reduced the proliferation of DLA and Ehrlich’s ascites carcinoma (EAC) cell lines, which were previously injected into the peritoneal cavities of healthy albino mice. A 50% growth inhibition was obtained at 7.80 μmol/L and 9.50 μmol/L, respectively, for the DLA and EAC cells, which was lower than that of the positive control, 5-fluorouracil (37.36 and 90.55 μmol/L). 

A new prenylated acridone alkaloid, *medica*cridone, and a new ferulate xanthone, *medica*xanthone, were identified for the first time in the methanol extract of a *C. medica* bark collected in Cameroon. The newly isolated compounds and the already known compounds, citracridone, 5-hydroxynoracronycine, citracridone-III, lichenxanthone, lichenxanthone, and atalantoflavone, were tested for their cytotoxic activity against the human prostate adenocarcinoma cell line PC-3, showing weak activity (IC_50_ from 60.5 to 80.0 μM) compared to that of the positive control, Doxorubicin (IC_50_ 0.9 μM) [37].

#### 3.4.4. Anti-Inflammatory and Analgesic Activity

The citron *C. medica* is rich in flavonoids, such as hesperidin, naringin, and apigenin, which possess strong anti-inflammatory activities [112]. The effect of the *C. medica* EO on nitric oxide (NO) production in lipopolysaccharide (LPS)-stimulated macrophages was tested by Mitropoulou et al. [77], who obtained rates of inhibition of NO production of 56% after 12 h and of 83% after 24 h at 0.063 mg/mL of EO. No significant inhibition of NO production was observed at the lowest concentration tested (50% after 12 h, and 80% after 24 h at 0.018 mg/mL). The EO obtained from the *C. medica cv*. Diamante peel, was instead able to reduce NO production in LPS-stimulated macrophages and possessed anti-inflammatory activity, with an IC_50_ value of 17.0 mg/mL, compared to indomethacin, which was used as a positive control (IC_50_ of 53.0 mg/mL) [92]. Flower and leaf extracts of the same cultivar showed an inhibitory effect on LPS-induced NO production in a macrophage RAW 264.7 cell line in a dose-dependent manner, although the IC_50_ levels were notably high (525.0 mg/mL and 574.0 mg/mL, respectively) [27]. The EO from fingered citron (*C. medica* L. var. Sarcodactylis) was able to reduce the production of the inflammatory cytokines tumor necrosis factor *α* (TNF-*α*), interleukin 1*β* (IL-1*β)*, and interleukin 6 (IL-6) in LPS-activated macrophages (RAW 264.7 cells) at 40% to 80% at the highest concentration (0.02%). The extract prevented the nuclear factor kappa-light-chain-enhancer of activated B cell (NF-kB) activation by inhibiting the nuclear factor of kappa light polypeptide gene enhancer in B-cell inhibitor, alpha (IkB-*α*) phosphorylation. Furthermore, the levels of phosphorylated mitogen-activated protein kinases (MAPKs: c-Jun NH2-terminal kinase, JNK, and extracellular-signal-regulated kinase, ERK) were significantly decreased after the pre-treatment of the LPS-stimulated cells [27]. Similar activity was shown by the fruit extract in HaCat cells stimulated by LPS [101]. A *C. medica*. var. Sarcodactylis Swingle fruit was included in a formulation of ten herbs, which was tested against systemic lupus erythematosus (SLE). The authors found that the formulation can inhibit the membrane-bound B-cell activating factor (mBAFF)-induced upregulation of BAFF and its receptor, BAFF-R, Bcl-2, interleukin 10 (IL-10), and NF-κB, in YAC-1 cells (isolated rat peripheral-blood lymphocytes); thus, it can be administrated in combination with glucocorticoids in order to reduce toxicity and to improve efficacy [100]. An in vivo application of the anti-inflammatory activity of a peel extract of *C. medica* was carried out by Sood et al. [113]. Wistar rats received an ethyl-acetate extract of *C. medica* peel (400 mg/kg) via oral administration, which caused an approximately twofold reduction in the volume of carrageenan-induced paw edema after 12 h. Subsequently, the ability of the *C. medica* peel extract to reduce paw edema was also investigated by Malleshappa et al. [24] by using ethanol as an extraction solvent. After 5 h, the ethanolic peel extract reduced the paw edema by 82.77 ± 0.88%, in a manner that was comparable to that of the standard utilized (indomethacin), which caused a reduction in volume of 88.62 ±1.16%. Therefore, although the results are not comparable, as they are expressed differently, both extracts showed anti-edematogenic activities in preclinical studies.

One of the common features of inflammatory responses is the development of pain [114]. Ethanol [24] and ethyl acetate [83] extracts of *C. medica* peel were also investigated for their potential analgesic effects using the hot-plate test. This method measures the response to an acute nociceptive stimulus by placing an animal on a heated surface. Both extracts showed significant analgesic activity against hyperalgesia induced by thermal stimuli. The oral administration of the ethanolic extract (400 mg/Kg) led an extension of the rats’ reaction times compared with the control (carrageenan-treated) group, as well as the ethyl-acetate extract (500 mg/Kg). No significant differences were observed compared to the diclofenac sodium (12.5 mg/Kg) control group. Regarding the carrageenan-induced paw-edema test, it was not possible to compare the results due to their different forms of expression.

#### 3.4.5. Other Activities

Few studies have reported the hypoglycemic activity of *C. medica* and its potential application in diabetes treatment. The enzymes *α*-amylase and *α*-glucosidase are implicated in the reduction in post-prandial hyperglycemia by retarding the adsorption of glucose [115], and the use of inhibitors of both enzymes represents a therapeutic strategy. Natural compounds possess a promising ability to regulate hyperglycemia via the downregulation of *α*-amylase and *α*-glucosidase enzymes, with fewer side effects than conventional drugs [116]. As stated in many studies, the *C. medica* isolated flavonoids apigenin and hesperetin could be the molecules responsible for its hypoglycemic activity [117], in addition to terpenoids, whose access to enzymatic sites may be facilitated by their lipophilicity [118]. The flowers, leaves, and fruits (endocarp and mesocarp) of *C. medica* cv Diamante at two maturation stages were investigated for their potential hypoglycemic effects by Menichini et al. [27]. All the extracts showed weaker inhibition of amylase and glucosidase enzymes than the control (acarbose: IC_50_ 50.0 ± 0.9 and 35.5 ± 1.2 µg/mL, respectively). The flowers inhibited enzyme activity, with IC_50_ > 1000 μg/mL, while the leaves were more active on the *α*-amylase (IC_50_ 438.5 ± 5.2 μg/mL) than on the *α*-glucosidase (IC_50_ 777.5 ± 5.4 μg/mL). The maturation stages affected the endocarp activity on the carbohydrate-hydrolyzing enzymes: the *α*-amylase was inhibited more by the mature fruits (IC_50_ 426.0 ± 4.4 μg/mL) than by the immature extract (IC_50_ 844.5 ± 3.6 μg/mL), while the opposite was observed for the *α*-glucosidase (IC_50_ 574.1 ± 5.8 μg/mL and IC_50_ 472.9 ± 4.7 μg/mL, respectively). The mesocarp extract showed higher IC_50_ values, which were indicative of lower inhibitory activity against both enzymes; no difference in *α*-amylase inhibition was observed, while, regarding the *α*-glucosidase, the immature fruit showed stronger activity. Peng et al. [53] suggested the use of *C. medica* var. Sarcodactylis fruit extracts in type 2 diabetes mellitus, reporting an insulin-secretagogue effect. In a preclinical study, *C. medica* cv Diamante hydroalcoholic peel extract significantly (*p* < 0.05) decreased the serum glucose level (187.8 ± 20.6 mg/dL, at 600 mg/kg) compared to that in the control group (282.9 ± 60.1 mg/dL) [33]. The 70% aqueous methanol extract from *C. medica* var. Etrog leaves decreased serum levels of glucose in a dose-dependent manner, with 105.2 ± 8.35 mg/dL and 87.4 ± 6.30 mg/dL at 200 and 400 mg/kg, respectively, compared to the standard Gliclazide (110.8 ± 7.24 mg/dL) and diabetic control groups (172.3 ± 82.09 mg/dL) [38]. Conforti et al. [50] demonstrated that *C. medica* cv. Diamante peel extract was able to inhibit the *α*-amylase enzyme (IC_50_ value of 625 µg/mL), which was significantly different from the control utilized (acarbose, IC_50_ of 50 ± 0.58 µg/mL). The same extract was found to inhibit the acetylcholinesterase activity with IC_50_ values of 621 ± 7.83 µg/mL, which was ascribed by the authors to the presence of monoterpenes. Cholinesterase inhibition was also investigated by Tundis et al. [92]. The effects of EO obtained by hydro-distillation, cold-pressing, and supercritical carbon-dioxide extraction on acetylcholinesterase and butyrylcholinesterase were examined: hydro-distillation showed the strongest inhibitory activity against both enzymes (IC_50_ values of 171.3 mg/mL and 154.6 mg/mL respectively). However, the IC_50_ of the standard molecule utilized was much higher (physostigmine IC_50_ values of 0.2 ± 0.004 2.4 ± 0.02 mg/mL). These activities are also attributable to the presence of flavonoids; Dehghan et al. [119] investigated the interactions of hesperidin, diosmin, rutin, and naringin with protein targets of Alzheimer’s, Parkinson’s, and Huntington’s diseases using computational drug-design methods. Further studies are required to investigate the pharmacokinetic profiles of these compounds to increase their absorption in brain tissue; their use could represent a strategy against neurodegenerative diseases if co-administered with conventional drugs to reduce their potential toxic effects. Furthermore, *C. medica* is also an important source of polysaccharides, which can have many biological properties. The biological activity of polysaccharides seems to be related to their water-solubility; moreover, the presence of arabinose, mannose, glucose, or galactose might be linked to its immunomodulatory activity [120]. This is the case with the new heteropolysaccharide, CMSPB80-1, isolated by Peng et al. [61], which showed an immunoregulatory activity by increasing the production of NO by RAW264.7 macrophages and splenocyte proliferation. The proliferation of splenocytes is also enhanced by the sulfate derivative (CMSPW90-M1) of the heteropolysaccharide CMSPW90-1, which, in addition, was able to increase the phagocytosis of RAW264.7 cells [62]. The same activity has been observed for the heteropolysaccharide, CMSPA90-1, isolated by Gao et al. [63]. Furthermore, FCp-3 is a water-soluble polysaccharide identified in *C. medica* fruit by He et al., who demonstrated how this polysaccharide increased the proliferation of both splenocytes and thymocytes, potentially suggesting a potential immunomodulatory activity [64]. A new type of arabinoxylan (CM-1) and a new type of galactoarabinan (CM-2) were isolated for the first time from a whole fruit and exhibited antiproliferative activities against cancer-cell lines and immunostimulatory properties by increasing the secretion of pro-inflammatory cytokines, such as TNF-α and IL-6 [65]. By contrast, the galactorhamnan polysaccharide, K-CMLP (mainly composed of rhamnose and galactose), exerted anti-inflammatory effects by diminishing the production of TNF-α and IL-6 [66].

## 4. Conclusions

Over the last decades, the use of plant-derived extracts has received increased attention due to concerns over the possible adverse health effects caused by the use of conventional medicine. This review summarizes the main chemical properties and biological activities, examining new research approaches to share the knowledge on the therapeutic and nutraceutical properties of *C. medica*. The limitations on the introduction of *C. medica* into medical practice in relation to the activities screened in this review are due to the scarcity of preclinical and clinical studies. Additional in vivo experiments are certainly required to better investigate the effects of this species on the entire organism. As highlighted in our research, the biological properties of *C. medica* can be ascribed to the presence of specific molecules, such as polyphenols, alkaloids, coumarins, and terpenes, and to macronutrients and micronutrients, such as carbohydrates, minerals, vitamins, and amino acids, which are endowed with properties that are beneficial for health. 

In conclusion, based on the present literature review, it is possible to assert that *C. medica* can be considered an excellent candidate for treating various pathologies, mainly related to inflammation, oxidative stress, and microbial infection. However, new studies are needed to maximize *C. medica*’s potential for human health.

## Figures and Tables

**Table 1:** Checklist for assessment of the risk of bias in pre-clinical studies [13,14].

Checklist for Assessment of Risks of Bias in Pre-Clinical Studies
Are the hypothesis and objective of the study clearly described?
Are the main outcomes to be measured clearly described?
Are the main findings of the study clearly described?
Are the samples size calculations reported?
Are the animals randomly housed during the experiment?
Are the investigators blinded from knowledge which treatment used?
Are the outcome assessors blinded?
Is the dose/route of administration of *Citrus medica* L. properly reported?
Is the dose/route of administration of the drug in co-treatment properly reported?
Is the frequency of treatments adequately described?

**Table 2:** Macronutrients, amino acids, minerals, and water-soluble vitamins identified in *C. medica* L.

Nutrient Compounds	Part of Plant	Quantitative	References
**Minerals**			
Calcium (Ca)	peel, pulp	107.39–195.91 mg/100 g FW	[17]
Copper (Cu)	peel, pulp	0.061–0.45 mg/100 g FW	[17]
Iron (Fe)	peel, pulp	0.82–2.92 mg/100 g FW	[17]
Magnesium (Mg)	peel, pulp	5.86–16.29 mg/100 g FW	[17]
Manganese (Mn)	peel, pulp	0.052–0.266 mg/100 g FW	[17]
Potassium (K)	peel, pulp	126.04–263.27 mg/100 g FW	[17]
Sodium (Na)	peel, pulp	6.74–27.92 mg/100 g FW	[17]
Zinc (Zn)	peel, pulp	0.24–0.51 mg/100 g FW	[17]
	**Vitamins**		
Ascorbic acid (vitamin C)	peel, pulp, exocarp, mesocarp, endocarp, seeds	0.23–2.39 mg/100 g FW	[17]
		2.33–7.95 mg/100 g DW	[15]
	fructus	11.61 ± 2.50 mg/100 g FW	[18]
	peel	-	[19]
	peel	-	[20]
	fructus	-	[21]
	juice	18.49 ± 0.52 mg/100 g FW	[3]
Niacin (vitamin B3)	peel, pulp	0.05–0.63 mg/100 g FW	[17]
Pyridoxine (vitamin B6)	peel, pulp	0.75–10.12 mg/100 g FW	[17]
Riboflavin (vitamin B2)	peel, pulp, exocarp, mesocarp, endocarp, seeds	0.37–1.16 mg/100 g FW	[17]
		1.85–6.38 mg/100 g DW	[15]
Thiamin (vitamin B1)	peel, pulp, exocarp, endocarp	1.32–3.65 mg/100 g FW	[17]
		0.18–0.40 mg/100 g DW	[15]
**Essential amino acids**			
Histidine	peel, pulp	7.68–38.04 mg/100 g FW	[17]
Isoleucine	peel, pulp	16.14–81.95 mg/100 g FW	[17]
Leucine	peel, pulp	30.05–126.24 mg/100 g FW	[17]
Lysine	peel, pulp	27.37–94.46 mg/100 g FW	[17]
		-	[22]
Methionine	peel, pulp	1.63–11.53 mg/100 g FW	[17]
Phenylalanine	peel, pulp, exocarp, endocarp, mesocarp, seeds	19.21–89.44 mg/100 g FW	[17]
		-	[15]
Threonine	albedo, pulp	-	[22]
Valine	peel, pulp, albedo, pulp	29.64–121.92 mg/100 g FW	[17]
		-	[22]
**Non-essential amino acids**			
Alanine	peel, pulp	57.55–153.99 mg/100 g FW	[17]
	albedo, pulp		[22]
Arginine	peel, pulp	18.64–90.62 mg/100 g FW	[17]
Asparagine	peel, oil glands, albedo, pulp	-	[22]
Aspartic acid	peel, pulp	232.86–637.32 mg/100 g FW	[17]
Cystine	peel, pulp	1.76–1.82 mg/100 g FW	[17]
Glutamic acid	peel, pulp	71.47–227.50 mg/100 g FW	[17]
Glycine	peel, pulp	21.15–108.48 mg/100 g FW	[17]
Proline	peel, pulp	55.22–150.18 mg/100 g FW	[17]
		-	[15]
		-	[22]
Serine	peel, pulp	22.45–78.84 mg/100 g FW	[17]
Tryptophan	exocarp, endocarp, mesocarp	-	[17]
Tyrosine	peel, pulp	12.51–53.74 mg/100 g FW	[17]
**Macronutrients**			
Moisture content	peel, pulp	81.78–86.03 g/100 g FW	[17]
Fat	peel, pulp	0.39–0.56 g/100 g FW	[17]
Protein	peel, pulp	0.80–2.99 g/100 g FW	[17]
Ash	peel, pulp	0.44–1.23 g/100 g FW	[17]
Carbohydrates	peel, pulp	9.19–16.60 g/100 g FW	[17]
Energy	peel, pulp	53.74–73.06 g/100 g FW	[17]
Glucose	peel, pulp	0.92–2.27 g/100 g FW	[17]
Fructose	peel, pulp	1.60–2.95 g/100 g FW	[17]
Sucrose	peel, pulp	0.27–1.03 g/100 g FW	[17]

**Table 3:** Flavonoids, phenolic acids, and neolignans identified in *C. medica* L.

Compounds	Formula	Structure	Extraction Method	Chemical Analysis	Part of the Plant	Quantitative	References
Apigenin	C_15_H_12_O_5_	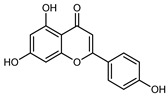	Maceration 70% EtOH	HPLC	flavedo	62.80 mg/kg FW	[33]
			Exhaustive maceration 70% EtOH	HPLC	flowers, leaves, mesocarp, endocarp	58.00–941.00 mg/kg FW	[27]
			Maceration 100% EtOH	UPLC–DAD	peel and pulp	24.26 ± 1.67 µg/g FW	[34]
Apigenin-6,8-di-*C*-glucoside	C_27_H_30_O_15_	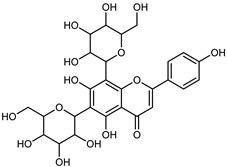	UAE 50%MeOH	HPLC–Q/TOF–MS	fructus	-	[35]
Atalantoflavon	C_21_H_18_O_4_	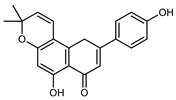	Maceration Acetone	COSY, NOESY, HMQC, HMBC, HR–ESI–MS	root bark, stem bark	-	[36]
			Maceration MeOH	ESI–HR, EI–MS, HMQC, HMBC	bark	-	[37]
Catechin	C_15_H_14_O_6_	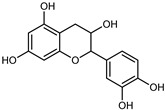	UAE EtOH 80%	UHPLC–QTOF–IMS	mesocarp, endocarp, seeds	5.14–57.87 mg/100 g DW	[15]
			Maceration 100% EtOH	UPLC–DAD	flavedo, pulp	4.34–68.78 µg/g FW	[34]
Dihydrokaem-pferide	C_16_H_14_O_6_	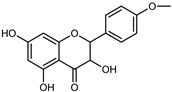	Maceration 70% MeOH	UV, MS, NMR	leaves	-	[38]
Dihydroquercetin	C_15_H_12_O_7_	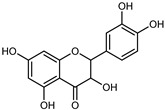	UAE EtOH 80%	UHPLC–QTOF–IMS	exocarp, endocarp, seeds	-	[15]
Epicatechin	C_15_H_14_O_6_	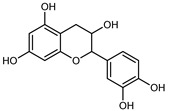	Maceration 100% EtOH	UPLC–DAD	flavedo, pulp	9.85–105.10 µg/g FW	[34]
Eriocitrin (Eriodictyol-7-*O*-rutinoside)	C_27_H_32_O_15_	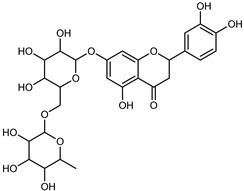	Maceration MeOH and 0.1% HCl	HPLC–PDA–MS	fructus	-	[39]
Herbacetin	C_15_H_10_O_7_	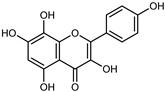	UAE EtOH 80%	UHPLC–QTOF–IMS	exocarp, mesocarp, seeds	-	[15]
Hesperetin	C_16_H_14_O_6_	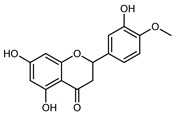	Dynamic maceration 70% EtOH	HPLC	flavedo	0.39–1.82 mg/g DW	[26]
			UAE EtOH 80%	UHPLC–QTOF–IMS	exocarp, endocarp, mesocarp, seeds	-	[15]
			Maceration 70% EtOH	HPLC	flavedo	50.4 mg/kg FW	[33]
			Exhaustive maceration 70% EtOH	HPLC	flowers, leaves, mesocarp, endocarp	203.80 mg/kg FW	[27]
Hesperetin-7-*O*-rutinoside	C_28_H_34_O_15_	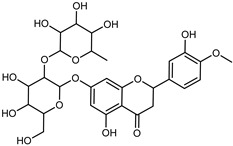	Maceration MeOH and 0.1% HCl	HPLC–PDA–MS	fructus	-	[39]
Hesperidin	C_28_H_34_O_15_	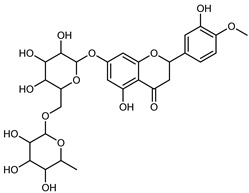	PLE MeOH	HPLC–DAD	fructus	30.36 µg/mL	[40]
			Dynamic maceration with 70% EtOH	HPLC	flavedo	1.86–2.77 mg/g DW	[26]
			UAE 80% EtOH	UHPLC–QTOF–IMS	exocarp, mesocarp, endocarp, seeds	383.02–3307.25 mg/100 g DW	[15]
			Exhaustive maceration 70% EtOH	HPLC	flowers, leaves, mesocarp, endocarp	9.00–224.30 mg/kg FW	[27]
			UAE 50% MeOH	HPLC–Q/TOF–MS	fructus	0.84–1.84 mg/g DW	[35]
Kaempferol 3-*O*-rutinoside	C_27_H_32_O_15_	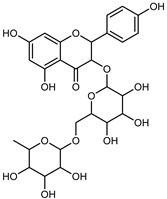	Dynamic maceration 70% EtOH	HPLC	flavedo	-	[26]
Limocitrol 3-*α*-l-arabinopyranosyl-(1->3) -galactoside	C_29_H_34_O_18_	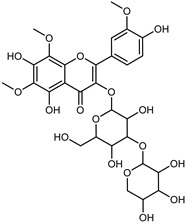	CPE 85% EtOH	UPLC–QTOF–MS/MS	fructus	-	[41]
Lonchocarpol A	C_25_H_28_O_5_	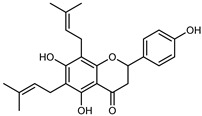	Maceration Acetone	COSY, NOESY, HMQC, HMBC, HR–ESI–MS	root bark, stem bark	-	[36]
Naringenin 7-*O*-glucoside	C_21_H_22_O_10_	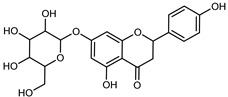	UAE 80% EtOH	UHPLC–QTOF–IMS	exocarp, mesocarp, seeds	-	[15]
Naringin	C_27_H_32_O_14_	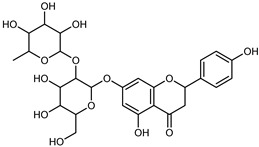	Exhaustive extraction 70% EtOH	HPLC	fructus	556.00 mg/kg FW	[27]
			UAE 80% EtOH	UHPLC–QTOF–IMS	exocarp, mesocarp, endocarp, seeds	36.82–295.15 mg/100 g DW	[15]
			UAE 80% EtOH	HPLC–QTOF–MS	fructus	0.43–0.61 mg/g DW	[35]
			Maceration 70% EtOH	HPLC	flavedo	18.60 mg/kg FW	[33]
Neodiosmin (Diosmetin-7-*O*-neoheseridoside)	C_28_H_32_O_15_	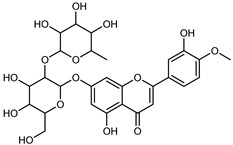	CPE 85% EtOH	UPLC–QTOF–MS/MS	fructus	-	[41]
Diosmin			Exhaustively maceration 70% EtOH	HPLC	flowers, leaves, mesocarp, endocarp	18.20–372.50 mg/kg FW	[27]
Neohesperidin (hesperetin-7-*O*-neohesperidoside)	C_28_H_34_O_15_	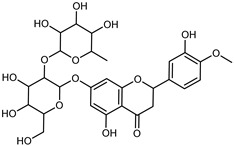	Maceration MeOH and 0.1% HCl	HPLC–PDA–MS	fructus	-	[39]
Nobiletin	C_21_H_22_O_8_	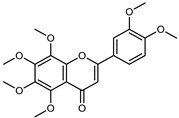	UAE EtOH 80%	UHPLC–QTOF–IMS	exocarp, mesocarp, endocarp, seeds	25.63–94.32 mg/100 g DW	[15]
Phloretin-3′, 5′-di-*C*-glucoside	C_27_H_34_O_15_	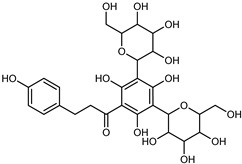	Maceration MeOH and 0.1%HCl	HPLC–PDA–MS	fructus	-	[39]
Quercetin	C_15_H_10_O_7_	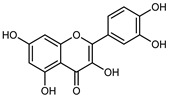	Soxhlet MeOH 65 °C	HPLC	fructus 2	0.025 mg/g DW	[42]
			Maceration 70% EtOH	HPLC	flavedo	18.20 mg/kg FW	[33]
			Dynamic maceration 70% EtOH	HPLC	flavedo	1.62–3.01 mg/g DW	[26]
			Exhaustive maceration 70% EtOH	HPLC	flowers, leaves, mesocarp, endocarp	11.00–580.80 mg/kg FW	[27]
Rutin	C_27_H_30_O_16_	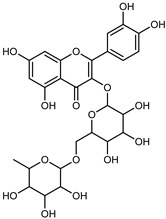	Maceration 100% EtOH	UPLC–DAD	flavedo and pulp	19.39–115.47 µg/g FW	[34]
			Dynamic maceration 70% EtOH	HPLC	flavedo	0.20–0.42 mg/g DW	[26]
			UAE EtOH 80%	UHPLC–QTOF–IMS	exocarp, mesocarp, endocarp	74.08–328.82 mg/100 g DW	[15]
			70% MeOH	UV, MS, NMR	leaves	-	[38]
Sakuranetin	C_16_H_14_O_5_	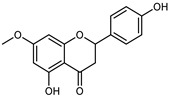	Maceration on cold 70% MeOH	UV, MS, NMR	leaves	-	[38]
Stachannin Scutellarein 4′-methyl ether 7-glucoside	C_22_H_22_0_11_	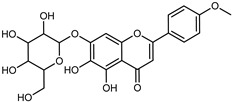	UAE EtOH 80%	UHPLC–QTOF–IMS	exocarp, endocarp, seeds	-	[15]
Tangeritin	C_20_H_20_O_7_	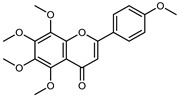	UAE EtOH 80%	UHPLC–QTOF–IMS	exocarp, mesocarp, endocarp, seeds	18.96–164.88 mg/100 g DW	[15]
Vitexin	C_21_H_20_0_10_	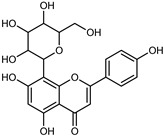	UAE EtOH 80%	UHPLC–QTOF–IMS	exocarp, endocarp, seeds	-	[15]
Vitexin-2-rhamnoside	C_27_H_30_O_14_	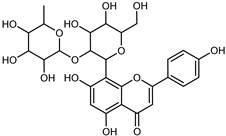	PLE MeOH	HPLC–DAD	fructus	-	[40]
3,5,6-Trihydroxy-3′,4′,7-trimethoxyflavone	C_18_H_16_O_8_	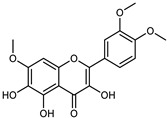	UAE EtOH 80%	UHPLC–QTOF–IMS	exocarp, mesocarp, endocarp, seeds	-	[15]
5,7-Dihydroxy-3′, 4′, 5′-trimethoxyflavone	C_18_H_16_O_7_	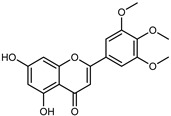	UAE EtOH 80%	UHPLC–QTOF–IMS	exocarp, seeds	-	[15]
5-Demethylnobiletin	C_20_H_20_O_8_	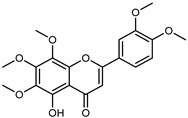	Dynamic maceration 70% EtOH	HPLC	flavedo	-	[26]
6,8-di-*C*-glucosyldiosmetin	C_28_H_32_0_16_	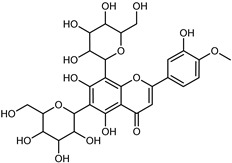	PLE MeOH	HPLC–DAD	fructus	13.51 µg/mL	[40]
7-*O*-Methyl-aromadendrin	C_16_H_14_O_6_	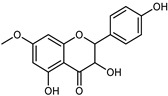	Maceration on cold 70% MeOH	UV, MS, NMR	leaves	-	[38]
Scoparin (Chrysoeriol 8-C-glucoside)	C_22_H_22_O_11_	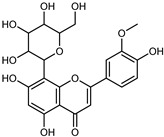	MeOH under reflux	EI–MS, HR–EI–MS	fresh fruit	-	[43]
Phenolic acids							
Benzoic acid	C_7_H_6_O_2_	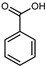	Soxhlet with MeOH	HPLC	fructus	0.00103 mg/g DW	[42]
Caffeic acid	C_9_H_8_O_4_	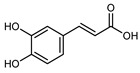	UAE EtOH 80%	UHPLC–QTOF–IMS	exocarp, mesocarp, endocarp, seeds	36.38–122.88 mg/100 g DW	[15]
			100% EtOH for 24 h	UPLC–DAD	flavedo, pulp	6.97–7.11 µg/g FW	[34]
Chlorogenic acid	C_16_H_18_O_9_	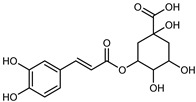	UAE EtOH 80%	UHPLC–QTOF–IMS	mesocarp, endocarp, seeds	66.66–109.85 mg/100 g DW	[15]
Gallic acid	C_7_H_6_0_5_	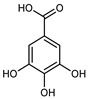	UAE EtOH 80%	UHPLC–QTOF–IMS	exocarp, mesocarp, endocarp	13.51–26.36 mg/100 g DW	[15]
			100% EtOH	UPLC–DAD	flavedo, pulp	16.84–39.02 µg/g FW	[34]
			Soxhlet with MeOH	HPLC	fructus	0.30 mg/g DW	[42]
*p*-Coumaric acid	C_9_H_8_0_3_	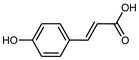	UAE EtOH 80%	UHPLC–QTOF–IMS	exocarp, mesocarp, endocarp, seeds	3.90–28.09 mg/100 g DW	[15]
Methyl-4-hydroxycinnamate	C_10_H_10_O_3_	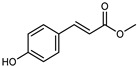	MeOH under reflux	EI–MS, HR–EI–MS	fresh fruit	-	[43]
Salicylic acid	C_7_H_6_O_3_	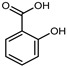	Soxhlet with MeOH	HPLC	fructus	0.16 mg/g DW	[42]
*trans*-Cinnamic acid	C_9_H_8_O_2_	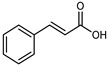	UAE EtOH 80%	UHPLC–QTOF–IMS	mesocarp, endocarp, seeds	0.42–13.06 mg/100 g DW	[15]
*trans*-Ferulic acid	C_10_H_10_O_4_	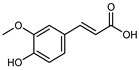	UAE EtOH 80%	UHPLC–QTOF–IMS	exocarp, mesocarp. Endocarp, seeds	19.85–96.79 mg/100 g DW	[15]
			100% EtOH	UPLC–DAD	flavedo and pulp	106.36–295.97 µg/g FW	[34]
			Dynamic maceration 70% EtOH	HPLC	flavedo	0.21–1.08 mg/g DW	[26]
Neolignans							
(7*E*)-1-Allyl alcohol-5,6-(11-isopropyl)-furanyl-3′,5′-dimethoxy-4′-glycerol-9′-isovalerate-3,4,7′,8′-benzodioxane neolignan	C_33_H_40_O_11_	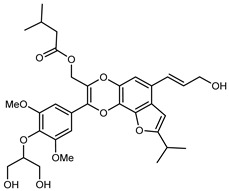	reflux 95% EtOH	NMR, HR–ESI–MS	fructus	-	[32]
2(7*E*,10′*E*,11*E*)-1-(9-Methoxyl)-propenyl-5-hydroxy-6-prenyl-8′-methylol-11′,16′-dihydroxy-15′,17′-dimethoxy-10′-phenylallyl alcohol-3,4,7′,8′-benzodioxane neolignan	C_35_H_38_O_10_	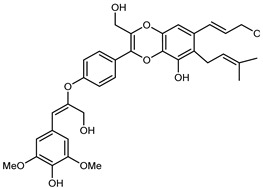	reflux 95% EtOH	NMR, HR–ESI–MS	fructus	-	[32]
(7*E*,11*E*)-1-(9-Methoxyl)-propenyl-5-hydroxy-6-geranyl-16′-hydroxy-15′,17′-dimethoxyphenyl-8′,11′-dimethylol-benzofuranyl 3,4,7′,8′-benzodioxane neolignan	C_39_H_44_O_11_	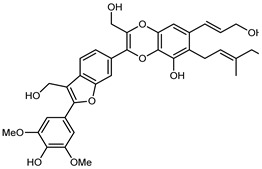	reflux 95% EtOH	NMR, HR–ESI–MS	fructus	-	[32]
1-(18,19-Dimethyl)-propanol-4-hydroxyl-5,6-(13-hydroxyl-12-methoxyl)-phenylethyl-7′-(4′-hydroxyl-5′-methoxy)-phenyl-9′-*O*-*β*-*D* glucopyranosyl-phenanthrofuran neolignan	C_36_H_42_O_13_	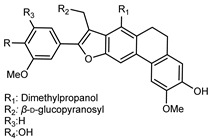	reflux 95% EtOH	NMR, HR–ESI–MS	fructus	-	[32]
1-(17-Furanyl)-ethyl-4-hydroxyl-5,6-(13-hydroxyl-12-methoxyl)- phenylethyl-7′-(3′,4′,5′-trimethoxy)-phenyl-9′-*O*-*β*-D-glucopyranosyl- phenanthrofuran neolignan	C_39_H_42_O_14_	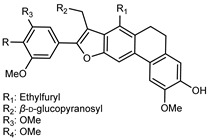	reflux 95% EtOH	NMR, HR–ESI–MS	fructus	-	[32]
1-(17*Z*)-Methyl-butanol-4-hydroxyl-5,6-(13-hydroxyl-12-methoxyl)- phenylethyl-7′-(4′-hydroxy-3′,5′-dimethoxy)-phenyl-9′-*O*-*β*-D-glucopyranosyl-phenanthrofuran neolignan	C_37_H_42_O_14_	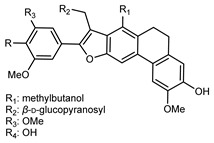	reflux 95% EtOH	NMR, HR–ESI–MS	fructus	-	[32]
(9′*E*)-4,5-(11,12-Dimethyl)- pyranyl-7′-(4′-hydroxy)- phenyl-4′-propenyl-8′-methylol-furanyl-6′-acetyl-1′,6-biphenyl-7-ketone	C_32_H_28_O_6_	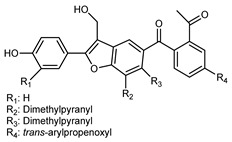	reflux 95% EtOH	NMR, HR–ESI–MS	fructus	-	[32]
(9*E*,9′*E*)-5-Isopentenyl-7′-(4′-hydroxy-5′-methoxy)-phenyl-4′-propenylketone-8′-methylol-furanyl-6′-acetyl-1′,6-biphenyl-7-ketone	C_33_H_30_O_8_	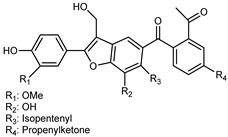	reflux 95% EtOH	NMR, HR–ESI–MS	fructus	-	[32]
(7E,10*E*)-4,5′-Dihydroxy-5-isopentenol-6-(7,8-trans allyl)-alcohol7′-(4′-hydroxy-3′,5′-dimethoxyl)-phenyl-9′,9′-dimethylol-1′,7′- bineolignan	C_34_H_34_O_11_	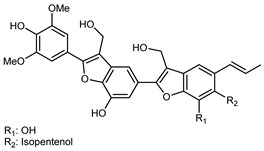	reflux 95% EtOH	NMR, HR–ESI–MS	fructus	-	[32]
(7*E*)-5′-Hydroxy-4,5-(13,14-dimethyl)-pyranyl-6-allyl alcohol-7′-(4′-hydroxy-3′,5′-dimethoxyl)-phenyl-9′,9′-dimethylol-1′,7′-bineolignan	C_34_H_32_O_10_	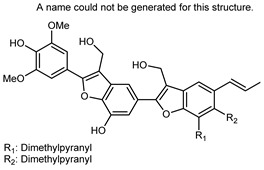	reflux 95% EtOH	NMR, HR–ESI–MS	fructus	-	[32]
(7*S*,8*R*)-9′,3-Dimethoxyl isoamericanol	C_20_H_22_O_7_	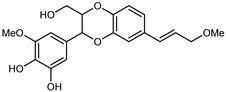	reflux 95% EtOH	NMR, HR–ESI–MS	fructus	-	[32]
(7*S*,8*R*,7′*S*,8′*R*)-7,8–7′,8′-*trans*-7′,8′-*Z*-Sesquiverniciasin A	C_27_H_25_O_9_	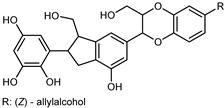	reflux 95% EtOH	NMR, HR–ESI–MS	fructus	-	[32]
(7*S*,8*R*,7′*S*,8′*R*)-7,8–7′,8′-*trans*-7′,8′-*E*-Sesquiverniciasin A	C_27_H_25_O_9_	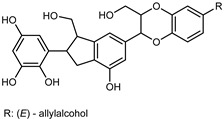	reflux 95% EtOH	NMR, HR–ESI–MS	fructus	-	[32]
Selamoellenin B	C_21_H_24_O_7_	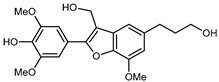	reflux 95% EtOH	NMR, HR–ESI–MS	fructus	-	[32]
Dendronbibisline A	C_30_H_26_O_7_	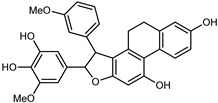	reflux 95% EtOH	NMR, HR–ESI–MS	fructus	-	[32]
Dendronbibisline B	C_25_H_24_O_7_	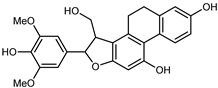	reflux 95% EtOH	NMR, HR–ESI–MS	fructus	-	[32]
Dendronbibisline C	C_32_H_32_O_8_	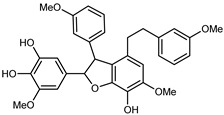	reflux 95% EtOH	NMR, HR–ESI–MS	fructus	-	[32]
Dendronbibisline D	C_33_H_34_O_8_	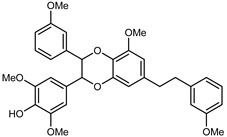	reflux 95% EtOH	NMR, HR–ESI–MS	fructus	-	[32]
Herpetiosol B	C_30_H_34_O_9_	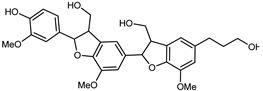	reflux 95% EtOH	NMR, HR–ESI–MS	fructus	-	[32]
Herpetosiols C	C_31_H_34_O_9_	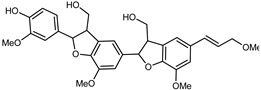	reflux 95% EtOH	NMR, HR–ESI–MS	fructus	-	[32]
Silychristin A	C_25_H_22_O_10_	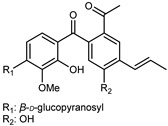	reflux 95% EtOH	NMR, HR–ESI–MS	fructus	-	[32]
Silychristin B	C_25_H_22_O_10_	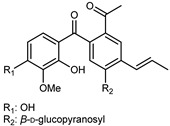	reflux 95% EtOH	NMR, HR–ESI–MS	fructus	-	[32]
(7*S*,8*R*)-*threo*-1′-[3′-Hydroxy-7-(4-hydroxy-3- methoxyphenyl)-8-hydroxymethyl-7,8-dihydrobenzofuran]acryl-aldehyde	C_19_H_18_O_6_	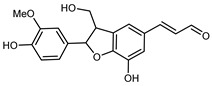	reflux 95% EtOH	NMR, HR–ESI–MS	fructus	-	[32]
(-)-(7*R*,8*S*,7′*E*)-4-Hydroxy-3,5,5′,9′-tetramethoxy-4′,7-epoxy-8,3′-neolign-7′-en-9-ol	C_22_H_26_O_7_	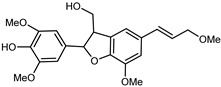	reflux 95% EtOH	NMR, HR–ESI–MS	fructus	-	[32]

**Table 4:** Terpenes identified in different parts of *Citrus medica* L.

Compounds	Formula	Structure	Extraction Method *	Analysis	Part of the Plant	Abundance	References
	**Monoterpenes**						
*ɑ*-Thujene	C_10_H_16_	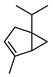	I	GC–MS	flavedo	0.2%	[50]
			II, III, IV	HRGC–MS	flavedo	0.28–0.59%	[49]
			V, VI, VII	GC–MS	flavedo	0.2–0.9%	[48]
			VIII	GC–MS–SPME	industrial essence	-	[57]
			X	GC–MS	fructus	1.20–1.29%	[53]
			XI	GC–MS	fructus	0.87%	[58]
			XII	HR–MAS–NMR	oil glands	-	[22]
			XV	GC–MS	exocarp, mesocarp	0.4–0.5%	[54]
*ɑ*-Thujone	C_10_H_16_O	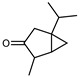	XIII	GC–MS	fresh fructus	4.29–5.05%	[45]
*Ꞵ*-Thujene	C_10_H_16_	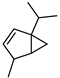	VIII	GC–MS–SPME	industrial essence	0.78%	[57]
*ɑ*-Pinene	C_10_H_16_	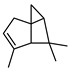	I	GC–MS	flavedo	0.49%	[50]
			II, III, IV	HRGC–MS	flavedo	0.69–1.46%	[49]
			V, VI, VII	GC–MS	flavedo	0.6–2.1%	[48]
			X	GC–MS	fructus	2.92–3.40%	[53]
			XI	GC–MS	fructus	1.99%	[58]
			XII	HR–MAS–NMR	oil glands	-	[22]
			XIII	GC–MS	fresh fructus	6.38–7.73%	[45]
			XV	GC–MS	exocarp, mesocarp	1.4–1.6%	[54]
Sabinene	C_10_H_16_	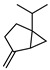	I	GC–MS	flavedo	0.64%	[50]
			II, III, IV	HRGC–MS	flavedo	0.14–0.22%	[49]
			V, VI, VII	GC–MS	flavedo	0.1–0.3%	[48]
			XII	HR–MAS–NMR	oil glands	-	[22]
Camphene	C_10_H_16_	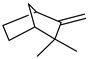	II, III, IV	HRGC–MS	flavedo	0.01%	[49]
			V, VI, VII	GC–MS	flavedo	trace	[48]
			VIII	GC–MS–SPME	industrial essence	0.04%	[57]
			X	GC–MS	fructus	0.02–0.03%	[53]
			XIII	GC–MS	fresh fruit	0.22–0.29%	[45]
*cis*-Sabinene hydrate	C_10_H_18_O	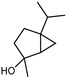	II, III, IV	HRGC–MS	flavedo	0.04–0.06%	[49]
			V, VI, VII	GC–MS	flavedo	trace	[48]
*trans*-Sabinene hydrate	C_10_H_18_O	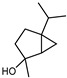	VIII	GC–MS–SPME	industrial essence	-	[57]
*Ꞵ*-Pinene	C_10_H_16_	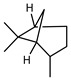	I	GC–MS	flavedo	0.63%	[50]
			II, III, IV	HRGC–MS	flavedo	0.69–1.47%	[49]
			V, VI, VII	GC–MS	flavedo	1.0–2.0%	[48]
			VIII	GC–MS–SPME	industrial essence	20.07%	[57]
			X	GC–MS	fructus	2.48–2.88%	[53]
			XI	GC–MS	fructus	2.02%	[58]
			XII	HR–MAS–NMR	flavedo, oil glands	-	[22]
			XIII	GC–MS	fresh fructus	2.64–3.18%	[45]
			XIV	GC–MS	exocarp, mesocarp	2.4–2.5%	[54]
Myrcene	C_10_H_16_	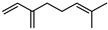	I	GC–MS	flavedo	0.89	[50]
			II, III, IV	HRGC–MS	flavedo	1.13–1.47%	[49]
			V, VI, VII	GC–MS	flavedo	0.8–1.6%	[48]
			XII	HR–MAS–NMR	oil glands	-	[22]
			VIII	GC–MS–SPME	industrial essence	2.24%	[57]
			X	GC–MS	fructus	1.64–1.76%	[53]
			XI	GC–MS	fructus	1.25%	[58]
Limonene	C_10_H_16_	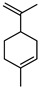	I	GC–MS	flavedo	15.20%	[50]
			II, III, IV	HRGC–MS	flavedo	25.70–60.30 g/100 g DW	[49]
			V, VI, VII	GC–MS	flavedo	34.6–60.8%	[48]
			VIII	GC–MS–SPME	industrial essence	41.07%	[57]
			X	GC–MS	fructus	51.24–57.63%	[53]
			XI	GC–MS	fructus	52.44%	[58]
			XII	HR–MAS–NMR	flavedo, oil glands, albedo	-	[22]
			XIII	GC–MS	fresh fructus	32.07–36.37%	[45]
			XIV	UHPLC–QTOF–IMS	exocarp, mesocarp, endocarp, seeds	-	[15]
			XV	GC–MS	exocarp, mesocarp	75.8–76.2%	[54]
Decane	C_10_H_22_	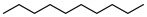	II, III, IV	HRGC–MS	flavedo	trace	[49]
Decanal	C_10_H_20_O	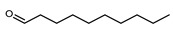	II, III, IV	HRGC–MS	flavedo	0.04–0.07	[49]
			V, VI, VII	GC–MS	flavedo	0.1%	[48]
			VIII	GC–MS–SPME	industrial essence	0.27%	[57]
Octyl acetate	C_10_H_20_O_2_	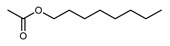	II, III, IV	HRGC–MS	flavedo	0.01%	[49]
			VIII	GC–MS	industrial essence	0.16%	[57]
Citronellol	C_10_H_20_O	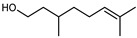	II, III, IV	HRGC–MS	flavedo	0.03–0.11%	[49]
*cis*-Limonene oxide	C_10_H_16_O	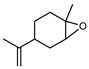	II, III, IV	HRGC–MS	flavedo	0.01%	[49]
			VIII	GC–MS–SPME	industrial essence	-	[57]
*trans*-Limonene oxide	C_10_H_16_O	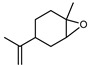	II, III, IV	HRGC–MS	flavedo	trace	[49]
			VIII	GC–MS	industrial essence	0.28%	[57]
*trans*-Carveol	C_10_H_16_O	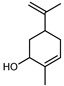	V, VI, VII	GC–MS	flavedo	0.1%	[48]
Carveol	C_10_H_16_O	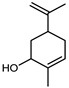	XV	GC–MS	mesocarp	0.1%	[54]
Camphor	C_10_H_16_O	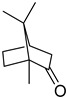	II, III, IV	HRGC–MS	flavedo	0.01%	[49]
Citronellal	C_10_H_18_O	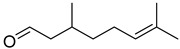	II, III, IV	HRGC–MS	flavedo	0.04–0.06%	[49]
			V, VI, VII	GC–MS	flavedo	0.1–0.2%	[48]
			VIII	GC–MS–SPME	industrial essence	0.27%	[57]
			XII	HR–MAS–NMR	oil glands	-	[22]
			XIII	GC–MS	fresh fructus	0.11%	[45]
Borneol	C_10_H_18_O	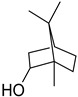	II, III, IV	HRGC–MS	flavedo	0.01%	[49]
(Z)-*Ꞵ*-Ocimene	C_10_H_16_	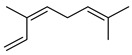	II, III, IV	HRGC–MS	flavedo	0.75–1.19%	[49]
			V, VI, VII	GC–MS	flavedo	1.4–1.5%	[48]
			VIII	GC–MS–SPME	industrial essence	-	[57]
			XI	GC–MS	fructus	0.94%	[58]
(E)-*Ꞵ*-Ocimene	C_10_H_16_	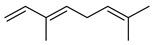	II, III, IV	HRGC–MS	flavedo	1.10–1.74%	[49]
			V, VI, VII	GC–MS	flavedo	1.9–2.1%	[48]
			VIII	GC–MS–SPME	industrial essence	0.07%	[57]
			XIII	GC–MS	fresh fructus	0.55–0.99%	[45]
			X	GC–MS	fructus	0.23–0.93%	[53]
			XI	GC–MS	fructus	0.65%	[58]
			XV	GC–MS	exocarp, mesocarp	1.1–1.2%	[54]
Citral	C_10_H_16_O	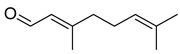	VIII	GC–MS–SPME	industrial essence	-	[57]
			X	GC–MS	fructus	1.96–2.34%	[53]
			XII	HR–MAS–NMR	flavedo	-	[22]
			XV	GC–MS	mesocarp	0.1%	[54]
Octanal	C_8_H_16_O	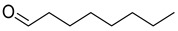	II, III, IV	HRGC–MS	flavedo	0.01%	[49]
			V, VI, VII	GC–MS	flavedo	-	[48]
			VIII	GC–MS–SPME	industrial essence	0.31%	[57]
*ɑ*-Phellandrene	C_10_H_16_	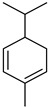	II, III, IV	HRGC–MS	flavedo	0.04–0.05%	[49]
			XV	GC–MS	mesocarp	trace	[54]
			V, VI, VII	GC–MS	flavedo	0.1%	[48]
			X	GC–MS	fructus	0.1%	[53]
*δ*-3-Carene	C_10_H_16_	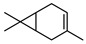	I	GC–MS	flavedo	2.30%	[50]
			II, III, IV	HRGC–MS	flavedo	trace	[49]
3-Carene	C_10_H_16_	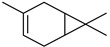	XIII	GC–MS	fresh fructus	8.15–9.01%	[45]
4-Carene	C_10_H_16_	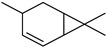	VIII	GC–MS–SPME	industrial essence	0.10%	[57]
*γ*-Terpinene	C_10_H_16_	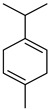	I	GC–MS	flavedo	10.27%	[50]
			II, III, IV	HRGC–MS	flavedo	21.19–23.44%	[49]
			V, VI, VII	GC–MS	flavedo	22.1–24.6%	[48]
			VIII	GC–MS–SPME	industrial essence	8.35%	[57]
			X	GC–MS	fructus	27.01–33.71%	[53]
			XI	GC–MS	fructus	28.41%	[58]
			XII	HR–MAS–NMR	flavedo, oil glands	-	[22]
			XIII	GC–MS	fresh fructus	22.44–25.23%	[45]
			XV	GC–MS	exocarp, mesocarp	15.0–16.5%	[54]
*ɑ*-Terpinene	C_10_H_16_	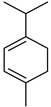	II, III, IV	HRGC–MS	flavedo	0.35–0.41%	[49]
			V, VI, VII	GC–MS	flavedo	trace	[48]
			X	GC–MS	fructus	1.28%	[53]
			XI	GC–MS	fructus	0.81%	[58]
Terpinolene	C_10_H_16_	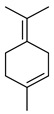	I	GC–MS	flavedo	0.91%	[50]
			II, III, IV	HRGC–MS	flavedo	0.87–1.00%	[49]
			V, VI, VII	GC–MS	flavedo	1.0–1.2%	[48]
			VIII	GC–MS–SPME	industrial essence	0.33%	[57]
			X	GC–MS	fructus	1.25–1.54%	[53]
			XIII	GC–MS	industrial essence	-	[45]
			XV	GC–MS	exocarp, mesocarp	0.2–0.6%	[54]
Linalool	C_10_H_18_O	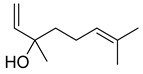	I	GC–MS	flavedo	1.15%	[50]
			II, III, IV	HRGC–MS	flavedo	0.10–0.20 g/100 g DW	[47]
			V, VI, VII	GC–MS	flavedo	0.1–0.3%	[48]
			VIII	GC–MS–SPME	industrial essence	1.73%	[57]
			XIII	GC–MS	fresh fructus	0.16–0.18%	[45]
Linalool oxide	C_10_H_18_O_2_	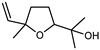	VIII	GC–MS–SPME	industrial essence	0.28%	[57]
Allocimene	C_10_H_16_	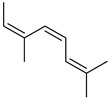	I	GC–MS	flavedo	0.70%	[50]
Terpinen-4-ol	C_10_H_18_O	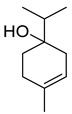	I	GC–MS	flavedo	1.02%	[50]
			II, III, IV	HRGC–MS	flavedo	0.04–0.06%	[49]
			V, VI, VII	GC–MS	flavedo	0.1–0.2%	[48]
			X	GC–MS	fructus	0.34–0.51%	[53]
			VIII	GC–MS–SPME	industrial essence	0.31%	[57]
			XIII	GC–MS	fresh fructus	0.69–0.88%	[45]
*ɑ*-Terpineol	C_10_H_18_O	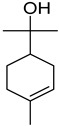	I	GC–MS	flavedo	2.64%	[50]
			V, VI, VII	GC–MS	flavedo	0.1–0.3%	[48]
			VIII	GC–MS–SPME	industrial essence	0.10%	[57]
			X	GC–MS	fructus	0.48–0.58%	[53]
			XIII	GC–MS	fresh fruit	1.17–1.61%	[45]
			XV	GC–MS	exocarp, mesocarp	0.1–0.4%	[54]
Nerol	C_10_H_18_O	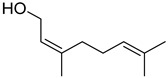	I	GC–MS	flavedo	4.69%	[50]
			V, VI, VII	GC–MS	flavedo	0.1–0.3%	[48]
			XIII	GC–MS	fresh fructus	0.9–1.53%	[45]
Neral	C_10_H_16_O	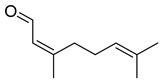	II, III, IV	HRGC–MS	flavedo	1.20–9.40 g/100 g DW	[49]
			V, VI, VII	GC–MS	flavedo	trace	[48]
			VIII	GC–MS–SPME	industrial essence	2.49%	[57]
			X	GC–MS	fructus	0.45%	[53]
			XII	HR–MAS–NMR	flavedo	-	[22]
			XIII	GC–MS	fresh fructus	1.04–1.60%	[45]
*p*-Cymen-8-ol	C_10_H_14_O	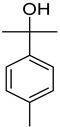	II, III, IV	HRGC–MS	flavedo	0.01%	[47]
*p*-Cymene	C_10_H_14_	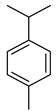	V, VI, VII	GC–MS	flavedo	0.4–0.6%	[48]
			XIV	GC–MS	exocarp, mesocarp	0.2–0.7%	[54]
			VIII	GC–MS–SPME	industrial essence	5.92%	[57]
			XIII	GC–MS	fresh fructus	1.64–2.77%	[45]
Geraniol	C_10_H_18_O	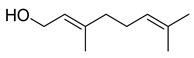	I	GC–MS	flavedo	4.63%	[50]
			II, III, IV	HRGC–MS	flavedo	0.10–8.50 g/100 g DW	[49]
			V, VI, VII	GC–MS	flavedo	0.1–0.7%	[48]
			X	GC–MS	fructus	0.55–0.58%	[53]
			VIII	GC–MS-SPME	industrial essence	0.27%	[57]
			XIII	GC–MS	fresh fructus	1.18–2.02%	[45]
Perillal	C_10_H_14_O	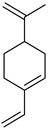	VIII	GC–MS–SPME	industrial essence	0.10%	[57]
Cuminol	C_10_H_14_O	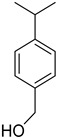	VIII	GC–MS–SPME	industrial essence	0.03%	[57]
Carvacrol	C_10_H_14_O	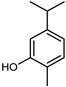	II, III, IV	HRGC–MS	flavedo	trace	[49]
Perilla aldehyde	C_10_H_14_O	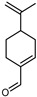	II, III, IV	HRGC–MS	flavedo	0.01–0.02%	[49]
**Sesquiterpenes**							
*δ*-Elemene	C_15_H_24_	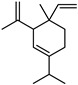	II, III, IV	HRGC–MS	flavedo	0.06–0.16%	[49]
			V, VI, VII	GC–MS	flavedo	0.1%	[48]
*Ꞵ*-Elemene	C_15_H_24_	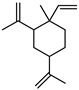	I	GC–MS	flavedo	0.1%	[50]
			II, III, IV	HRGC–MS	flavedo	0.1%	[49]
			V, VI, VII	GC–MS	flavedo	0.1%	[48]
			X	GC–MS	flavedo	0.1%	[53]
Copaene	C_15_H_24_	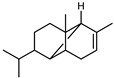	X	GC–MS	fructus	0.02%	[53]
*trans*-Caryophyllene	C_15_H_24_	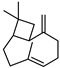	I	GC–MS	flavedo	0.41%	[50]
*ɑ*-Bisabolol	C_15_H_26_O	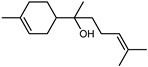	V, VI, VII	GC–MS	flavedo	0.2%	[48]
*ɑ*-Bergamotene	C_15_H_24_	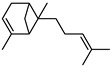	I	GC–MS	flavedo	1.09%	[50]
			XV	GC–MS	exocarp, mesocarp	0.3–0.6%	[54]
			X	GC–MS	fructus	0.07%	[53]
			V, VI, VII	GC–MS	flavedo	0.2–1.7%	[48]
*ɑ*-Himachalene	C_15_H_24_	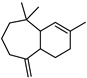	XV	GC–MS	exocarp, mesocarp	0.1–0.6%	[54]
*γ*-Gurjunene	C_15_H_24_	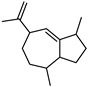	XV	GC–MS	mesocarp	trace	[54]
*ɑ*-Humulene	C_15_H_24_	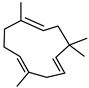	I	GC–MS	flavedo	0.13%	[50]
			II, III, IV	HRGC–MS	flavedo	0.1%	[49]
			V, VI, VII	GC–MS	flavedo	0.1%	[48]
			X	GC–MS	fructus	-	[53]
(*Z*)-*β*-Farnesene	C_15_H_24_	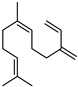	I	GC–MS	flavedo	0.14%	[50]
			II, III, IV	HRGC–MS	flavedo	trace	[49]
			V, VI, VII	GC–MS	flavedo	0.1%	[48]
*α*-Bisabolene	C_15_H_24_	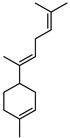	I	GC–MS	flavedo	0.10%	[50]
			IX	GC–MS	leaves	-	[59]
*β*-Bisabolene	C_15_H_24_	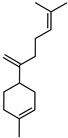	I	GC–MS	flavedo	1.39%	[50]
			II, III, IV	HRGC–MS	flavedo	0.03–0.05%	[49]
			V, VI, VII	GC–MS	flavedo	0.2–2.6%	[48]
			VIII	GC–MS–SPME	industrial essence	0.30%	[57]
Spathulenol	C_15_H_24_O	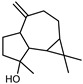	I	GC–MS	flavedo	0.1%	[50]
			V, VI, VII	GC–MS	flavedo	0.1%	[48]
*α*-*cis*-Bergamotene	C_15_H_24_	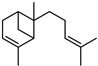	II, III, IV	HRGC–MS	flavedo	0.02–0.03%	[49]
*E*-*β*-Caryophyllene	C_15_H_24_	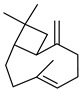	II, III, IV	HRGC–MS	flavedo	0.10 g/100 g DW	[49]
			VIII	GC–MS	industrial essence	0.23%	[57]
			IX	GC–MS	leaves	-	[59]
			X	GC–MS	fructus	0.06%	[53]
			XIII	GC–MS	fresh fructus	0.27–0.46%	[45]
			XIV	UHPLC–QTOF–IMS	mesocarp	-	[15]
*α*-*trans*-Bergamotene	C_15_H_24_	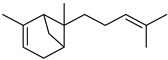	II, III, IV	HRGC–MS	flavedo	0.29–0.45%	[49]
			V, VI, VII	GC–MS	flavedo	0.2–1.7%	[48]
			IX	GC–MS	leaves	-	[59]
(*E*)-*β*-Farnesene	C_15_H_24_	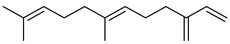	II, III, IV	HRGC–MS	flavedo	trace	[49]
			XV	GC–MS	exocarp	0.2%	[54]
			IX	GC–MS	leaves	-	[59]
(*Z*)-*β*-Santalene	C_15_H_24_	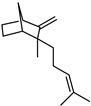	II, III, IV	HRGC–MS	flavedo	0.01%	[49]
Valencene	C_15_H_24_	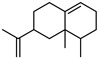	II, III, IV	HRGC–MS	flavedo	0.03–0.07%	[49]
Bicyclogermacrene	C_15_H_24_	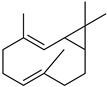	II, III, IV	HRGC–MS	flavedo	0.03–0.04%	[49]
(Z)-*α*-Bisabolene	C_15_H_24_	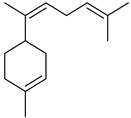	II, III, IV	HRGC–MS	flavedo	0.03–0.05%	[49]
*β*-Cadinene	C_15_H_24_	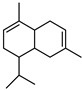	XIII	GC–MS	fresh fructus	0.74–1.09%	[45]
*α*-Cedrene	C_15_H_24_	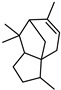	XIII	GC–MS	fresh fructus	0.55–0.64%	[45]
(*E*,*E*)-*α*-Farnesene	C_15_H_24_	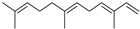	V, VI, VII	GC–MS	flavedo	trace	[48]
(*Z*)-*α*-Farnesene	C_15_H_24_	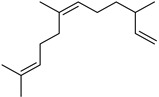	XV	GC–MS	exocarp	0.6%	[54]
*α*-Farnesene	C_15_H_24_	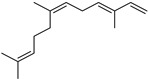	X	GC–MS	fructus	0.1%	[53]
			XV	GC–MS	mesocarp	0.2%	[54]
(*Z*)-*γ*-Bisabolene	C_15_H_24_	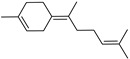	II, III, IV	HRGC–MS	flavedo	trace	[49]
Germacrene B	C_15_H_24_	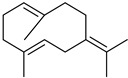	V, VI, VII	GC–MS	flavedo	0.1–0.3%	[48]
			X	GC–MS	fructus	-	[53]
Gemacrene D	C_15_H_24_	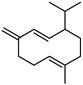	IX	GC–MS	leaves	-	[59]
			X	GC–MS	fructus	0.15–0.19%	[53]
Bicyclogermacrene	C_15_H_24_	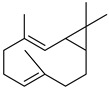	IX	GC–MS	leaves	-	[59]
			X	GC–MS	fructus	0.06%	[53]
(*E*)-Nerolidol	C_15_H_26_O	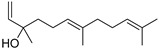	V, VI, VII	GC–MS	flavedo	0.1–0.3%	[48]
			IX	GC–MS	leaves	-	[59]
*Β*-Bisabolene	C_15_H_24_	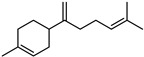	II, III, IV	HRGC–MS	flavedo	0.40–0.67%	[49]
Farnesol	C_15_H_26_O	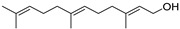	V, VI, VII	GC–MS	flavedo	trace	[48]
Farnesal	C_15_H_24_O	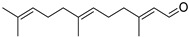	V, VI, VII	GC–MS	flavedo	trace	[48]
**Triterpenoids (Limonoids)**							
Limonyl acetate	C_28_H_34_O_9_	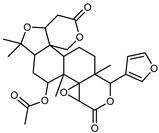	XIV	UHPLC–QTOF–IMS	exocarp, seeds	-	[15]
Limonin	C_26_H_30_O_8_	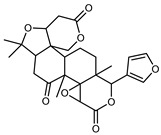	XVII	HPLC	citron waste	3.08 mg/100 g DW	[56]
			XVI	EI–MS, HR–EI–MS	fresh fructus	-	[43]
			XIV	HPLC–Q/TOF–MS	fructus	0.45–0.86 mg/g DW	[35]
			XVIII	ESI–HR, EI–MS, HMQC, HMBC	bark	-	[37]
Nomilin	C_28_H_34_O_9_	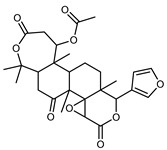	XVII	HPLC	citron waste	0.87 mg/100 g DW	[56]
			XVI	EI–MS, HR–EI–MS	fresh fructus	-	[43]
			XIV	HPLC–Q/TOF–MS	fructus	1.97–3.84 mg/g DW	[35]
Citrusin	C_28_H_34_O_11_	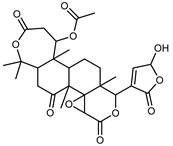	XVI	EI–MS, HR–EI–MS	fresh fructus	-	[43]
Obacunone	C_26_H_30_O_7_	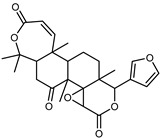	XVI	EI–MS, HR–EI–MS	fresh fructus	-	[43]
			XIV	HPLC–Q/TOF–MS	fructus	0.15–0.36 mg/g DW	[35]
Nomilinic acid	C_28_H_36_O_10_	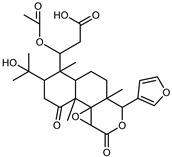	XIV	UHPLC–QTOF–IMS	exocarp, seeds	-	[15]
**Terpenoids**							
Geranyl acetate	C_12_H_20_O_2_	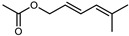	I	GC–MS	flavedo	0.75%	[50]
Citronellyl acetate	C_12_H_22_O_2_	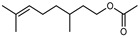	II, III, IV	HRGC–MS	flavedo	0.10 g/100 g DW	[49]
			V, VI, VII	GC–MS	flavedo	0.1–0.2%	[48]
Dihydrolinalyl acetate	C_12_H_22_O_2_	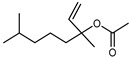	II, III, IV	HRGC–MS	flavedo	trace	[49]
*β*-lonone	C_13_H_20_O	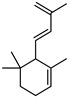	XIII	GC–MS	fresh fructus	0.20–0.49%	[45]
Linalyl acetate	C_12_H_20_O_2_	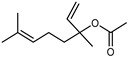	VIII	GC–MS	industrial essence	1.82%	[57]

* Essential oil extraction methods. I: Maceration of peel with *n*-hexane at room temperature; II: aspiration of the oil from the utricles present on the peel by means of a syringe with a thin needle; III: the rinds of the fruit were squeezed to cause the breaking of the utricles in order to release the oil, which was collected by extraction with hexane; IV: manual abrasion of the rind by means of a stainless-steel grater, followed by manual pressing and centrifugation of the water-oil emulsion; V: hydro-distillation; VI: soxhlet apparatus using pentane and ethanol as solvents; VII: SCF–CO_2_; VIII: alcoholic and industrial extraction method; IX: *n*-Hexanol was added to leaf powder; X: steam distillation; XI: distillation using a Clevenger-type apparatus; XII: the content of oil glands was obtained cutting the most superficial layer of flavedo to open oil glands; XIII: steam hydro-distillation; XIV: UAE; XV: exocarp and mesocarp were pulverized in liquid nitrogen with a chilled mortar and pestle, and then weighed and placed in MeOH. The mixtures were sonicated; XVI: MeOH under reflux; XVII: enzymatic treatment. XVIII: maceration in MeOH.

**Table 5:** Coumarins identified in various parts of *C. medica* L.

Compounds	Formula	Structure	Extraction Method	Method Analyses	Part of the Plant	Quantitative	References
Oxypeucedanin hydrate	C_16_H_16_O_6_	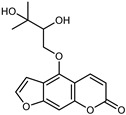	MeOH under reflux	EI–MS, HR–EI–MS	fresh fruit	2.03–21.30 g/100 g DW	[43]
Scoparone (6,7-dimethoxycoumarin)	C_11_H_10_O_4_	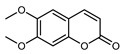	MeOH under reflux	EI–MS, HR–EI–MS	fresh fruit	-	[43]
			PLE MeOH	HPLCDAD	fructus	38.79 µg/mL	[40]
			UAE	HPLC–Q/TOF–MS	fructus	-	[35]
Skimmin	C_15_H_16_O_8_	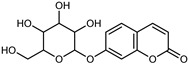	MeOH under reflux	EI–MS, HR–EI–MS	fresh fruit	-	[43]
Haploperoside A	C_22_H_28_O_13_	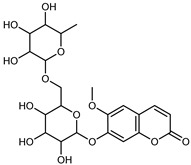	MeOH under reflux	EI–MS, HR–EI–MS	fresh fruit	-	[43]
Leptodactylone	C_11_H_10_O_5_	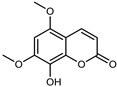	MeOH under reflux	EI–MS, HR–EI–MS	fresh fruit	-	[43]
Herniarin (7-methoycoumarin)	C_10_H_8_O_3_	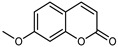	MeOH under reflux	EI–MS, HR–EI–MS	fresh fruit	-	[43]
Isomeranzin	C_15_H_16_O_4_	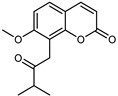	UAE	HPLC–Q/TOF–MS	fructus	-	[35]
Scopoletin	C_10_H_8_O_4_	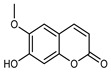	MeOH under reflux	EI–MS, HR–EI–MS	fresh fruit	-	[43]
			PLE MeOH	HPLC–DAD	fructus	53.56 µg/mL	[40]
Isoscopoletin	C_10_H_8_O_4_	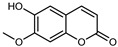	PLE MeOH	HPLC–DAD	fructus	63.06 µg/mL	[40]
Umbelliferone (7-hydroxycoumarin)	C_9_H_6_O_3_	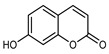	MeOH under reflux	EI-MS, HR–EI–MS	fresh fruit	-	[43]
			PLE MeOH	HPLC–DAD	fructus	40.23 µg/mL	[40]
Nordentatin	C_19_H_20_O_4_	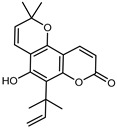	MeOH under reflux	EI–MS, HR–EI–MS	fresh fruit	-	[43]
			Maceration in acetone	COSY, NOESY, HMQC, HMBC, HR–ESI–MS	root bark, stem bark	-	[36]
2-pyrone	C_5_H_4_O_2_	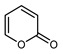	Maceration and UAE MeOH	GC–MS	exocarp, mesocarp	23.4–33.1%	[54]
Citropten (5,7-dimethoxycoumarin)	C_11_H_10_O_4_	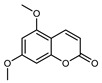	PLE MeOH	HPLC–DAD	fructus	106.47 µg/mL	[40]
			MeOH under reflux	HR–EI–MS1	fresh fruits	0.16–0.45 mg/g DW	[43]
			Maceration of peel with *n*-hexane at room temperature	GC–MS	fructus	12.64%	[50]
			UAE	HPLC–Q/TOF–MS	fructus	0.18–0.45 mg/g DW	[35]
Bergapten	C_12_H_8_O_4_	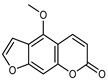	PLE MeOH	HPLC–DAD	fructus	35.07 µg/mL	[40]
			UAE	HPLC–Q/TOF–MS	fructus	-	[35]
			Maceration MeOH	ESI–HR, EI–MS, HMQC, HMBC	bark	-	[37]
Citrumedin-B	C_24_H_28_O_4_	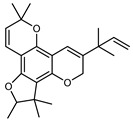	Acetone at room temperature	COSY, NOESY, HMQC, HMBC, HR–ESI–MS	root bark, stem bark	-	[36]
Xanthyletin	C_14_H_12_O_3_	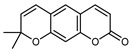	PLE MeOH	HPLC–DAD	fructus	-	[40]
			UAE with CHCl_3_	MEKC (micellar electrokinetic capillary chromatography)	root bark	-	[60]
Xanthoxyletin	C_15_H_14_O_4_	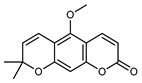	UAE with CHCl_3_	MEKC (micellar electrokinetic capillary chromatography)	root bark	-	[60]
5,8-dimethoxhypsoralene	C_12_H_8_O_4_	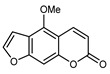	Maceration MeOH	ESI–HR, EI–MS, HMQC, HMBC	bark	-	[37]

**Table 6:** Other compounds identified in *C. medica* L.

Compounds	Formula	Structure	* Extraction Method	Method Analyses	Part of the Plant	Abundance	References
**Alkaloids**							
1,2,3,4-Tetrahydro-beta-carboline-3-carboxylic acid	C_12_H_12_N_2_O_2_	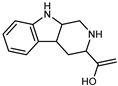	IX	EI–MS, HR–EI–MS	fresh fruit	-	[43]
**Acridine derivatives**							
*Medica*cridone	C_20_H_21_NO_4_	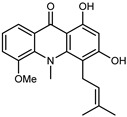	VIII	ESI–HR, EI–MS, HMQC, HMBC	bark	-	[37]
Citracridone-I	C_20_H_19_NO_5_	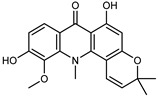	VIII	ESI–HR, EI–MS, HMQC, HMBC	bark	-	[37]
citracridone-III	C_19_H_17_NO_5_	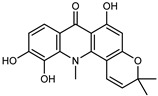	VIII	ESI–HR, EI–MS, HMQC, HMBC	bark	-	[37]
5-hydroxynoracronycine 3	C_19_H_17_NO_4_	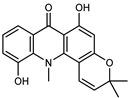	VIII	ESI–HR, EI–MS, HMQC, HMBC	bark	-	[37]
**Xanthones**							
*Medica*xanthone	C_51_H_75_O_8_	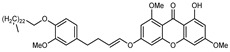	VIII	ESI–HR, EI–MS, HMQC, HMBC	bark	-	[37]
Lichenxanthone	C_15_H_12_O_6_	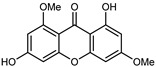	VIII	ESI–HR, EI–MS, HMQC, HMBC	bark	-	[37]
**Glycol**							
2,3-Butanediol	C_4_H_10_O_2_	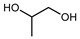	X	GC–MS	exocarp, mesocarp	23.7%	[54]
**Furan derivatives**							
Furfural	C_5_H_4_O_2_	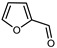	X	GC–MS	exocarp, mesocarp	3.9%	[54]
2(3*H*)-Furanone, 5-methyl	C_8_H_12_O_2_	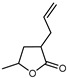	X	GC–MS	exocarp, mesocarp	0.9%	[54]
5- 5-Hydroxymethylfurfural	C_6_H_6_O_3_	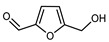	X	GC–MS	exocarp, mesocarp	1.9%	[54]
**Hydrocarbons**							
1,3-Cyclopentadiene	C_5_H_6_	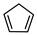	XIII	GC–MS	fresh fructus	1.75–2.36%	[45]
Benzene	C_6_H_6_	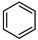	XI	GC–MS	fructus	1.67%	[58]
Eicosane	C_20_H_42_	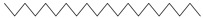	I	GC–MS	flavedo	0.10%	[50]
Nonacosane	C_29_H_60_	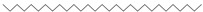	I	GC–MS	flavedo	0.10%	[50]
**Mono or polyunsaturated aldehyde**							
Undecanal	C_11_H_22_O	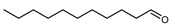	V, VI, VII	GC–MS	flavedo	0.1–0.2%	[48]
			II, III, IV	HRGC–MS	flavedo	0.03–0.06%	[49]
Dodecanal	C_12_H_24_O	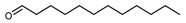	II, III, IV	HRGC–MS	flavedo	0.02–0.03%	[49]
			V, VI, VII	GC–MS	flavedo	0.1%	[48]
9,17-octadecadienal	C_18_H_32_O	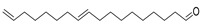	I	GC–MS	flavedo	9.29%	[50]
16-Octadecenal	C_18_H_34_O	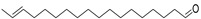	I	GC–MS	flavedo	0.10%	[50]
Nonanal	C_9_H_18_O	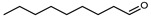	II, III, IV	HRGC–MS	flavedo	0.04–0.07%	[49]
Tetradecanal	C_14_H_28_O	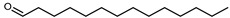	V, VI, VII	GC–MS	flavedo	0.1%	[48]
Pentadecanal	C_15_H_30_O	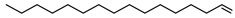	V, VI, VII	GC–MS	flavedo	0.1%	[48]
**Phenylpropanoids**							
Coniferin	C_16_H_22_O_8_	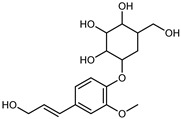	IX	EI–MS, HR–EI–MS	fresh fruit	-	[43]
Syringin	C_17_H_24_O_9_	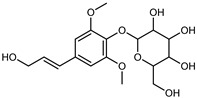	IX	EI–MS, HR–EI-MS	fresh fruit	-	[43]
**Phytosterols**							
Lupeol	C_26_H_32_O_7_	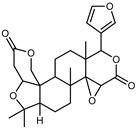	VIII	ESI–HR, EI–MS, HMQC, HMBC	bark	-	[37]
Stigmasterol	C_29_H_48_O	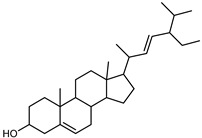	VIII	ESI–HR, EI–MS, HMQC, HMBC	bark	-	[37]
*Ꞵ*-Sitosterol	C_29_H_50_O	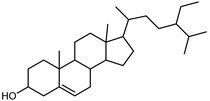	VIII	ESI–HR, EI–MS, HMQC, HMBC	bark	-	[37]
**Fatty acids and their esters**							
Lauric acid	C_12_H_24_O_2_	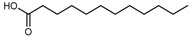	I	GC–MS	flavedo	0.11%	[50]
Myristic acid	C_14_H_28_O_2_	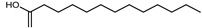	I	GC–MS	flavedo	0.23%	[50]
Palmitic acid	C_16_H_32_O_2_	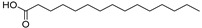	I	GC–MS	flavedo	5.17%	[50]
Hexadecanal	C_16_H_32_O	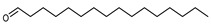	XIII	GC–MS	mesocarp	1.6%	[54]
			V, VI, VII	GC–MS	flavedo	0.1%	[48]
Pentadecanoic acid methyl ester	C_16_H_32_O_2_	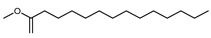	I	GC–MS	flavedo	0.22%	[50]
Palmitoleic acid	C_17_H_32_O_2_	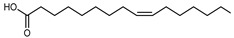	I	GC–MS	flavedo	0.19%	[50]
Heptadecanoic acid	C_17_H_34_O_2_	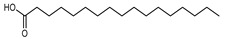	I	GC–MS	flavedo	0.20%	[50]
Stearic acid	C_18_H_36_O_2_	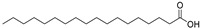	I	GC–MS	flavedo	0.18%	[50]
16-Octadecenal	C_18_H_34_O	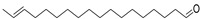	I	GC–MS	flavedo	0.10%	[50]
Linoleic acid, methyl ester	C_19_H_34_O_2_	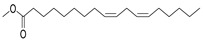	I	GC–MS	flavedo	0.19%	[50]
Linolenic acid, methyl ester	C_19_H_32_O_2_	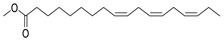	I	GC–MS	flavedo	0.41%0.30%	[50]
Stearic acid, methyl ester	C_19_H_38_O_2_	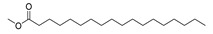	I	GC–MS	flavedo	0.30%	[50]
**Benzoates**							
Methyl vanillate methyl ester	C_9_H_10_O_4_	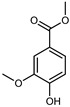	IX	EI–MS, HR–EI–MS	fresh fruit	-	[43]
Methyl benzoate	C_8_H_8_O_2_	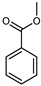	IX	EI–MS, HR–EI–MS	fresh fruit	-	[43]
Methyl paraben	C_8_H_8_O_3_	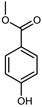	IX	EI–MS, HR–EI–MS	fresh fruit	-	[43]

* Extraction method. I: Maceration of peel with n-hexane at room temperature; II: aspiration of the oil from the utricles present on the peel by means of a syringe with a thin needle; III: the rinds of the fruit were squeezed to cause the breaking of the utricles in order to release the oil, which was collected by extraction with hexane; IV: manual abrasion of the rind by means of a stainless steel grater, followed by manual pressing and centrifugation of the water–oil emulsion; V: hydro-distillation; VI: soxhlet apparatus using pentane and ethanol as solvents; VII: SCF–CO2; VIII: maceration in MeOH; IX: MeOH under reflux; X: maceration and UAE in MeOH; XI: distillation using a Clevenger-type apparatus; XII: steam-based hydro-distillation; XIII: exocarp and mesocarp were pulverized in liquid nitrogen with a chilled mortar and pestle, and then weighed and placed in MeOH. The mixtures were sonicated.

**Table 7:** New polysaccharides isolated from *C. medica* L.

Compounds	Molecular Weight	Extraction Method	Method Analyses	Part of the Plant	Abundance	References
			**Polysaccharides**			
CMSPB80-1	103 kDa	Alkali extraction	HPGPC, FT–IR, methylation analysis, GC–MS, NMR	fructus	-	[61]
CMSPW90-1	18.8 kDa	Hot water	HPGPC, FT–IR, methylation analysis, GC–MS, NMR	pulp	-	[62]
CMSPW90-M1	75.4 kDa	Hot water	HPGPC, FT–IR, methylation analysis, GC–MS, NMR	pulp	-	[62]
CMSPA90-1	17.6 kDa	Acid extraction	HPGPC, FT–IR, methylation analysis, GC–MS, NMR	fructus	97.77% ± 1.4% (*w*/*w*) DW	[63]
FCp-1, FCp-2, FCp-3, and FCp-4	113.9, 32.6, 140.3, and 177.1 kDa respectively	Hot water	acid hydrolysis, methylation, IR, GC–MS, and NMR	fructus	-	[64]
CM-1 and CM-2	21.520 kDa, 22.303 kDa respectively	Hot water	Monosaccharide composition, linkage, and NMR	fructus	-	[65]
K-CLMP	3.76 × 10^3^ kDa	Hot water	methylation analysis, NMR	fructus	5.81%	[66]
Crude polysaccharides (FCPs)	-	Hot water	FT–IR	fructus	3.19 ± 0.10%	[67]

**Table 8:** Biological activities of *C. medica* L.

Test/Model	Concentration/Dosage Tested/Results	Part of the Plant	Extraction Method	Reference
**Antioxidant activity**				
ABTS	RSA 87.94% 0.2 mg/mL	fructus	CPE	[41]
DPPH	RSA 89.86% at 0.8 mg/mL			
ORAC	928.64 µmol TE/g			
DPPH	112.18 μg ascorbic acid/ mL	fructus	Soxhlet	[42]
NO	112.18 μg ascorbic acid/ mL			
TPC	177.50 ± 4.95 mgGAE/g			
TFC	165.52 ± 0.65 mgQUE/g			
TPC	227.45 ± 1.04 mg GAE/100 g FW	peel	MAE	[17]
	88.76 ± 1.38 mg GAE/100 g FW	pulp		
DPPH	22.79 ± 0.12 IC50 μg GAE/mL	peel		
	22.79 ± 0.12 IC50 μg GAE/mL	pulp		
ABTS	214.81 ± 1.45 mg TE/100 g FW	peel		
	71.53 ± 0.84 mg TE/100 g FW	pulp		
DPPH	EC_50_ 827.26 µg/mL	peel	Maceration	[20]
TPC	66.36 μg GAE/mg	peel		
	51.21 μg GAE/mg	pulp		
TFC	40.17μg cathecol equivalent/mg	peel		
	37.9μg cathecol equivalent/mg	pulp		
DPPH	0.80 ± 0.07 (IC50 mg/mL)	peel	Maceration	[33]
ABTS	3.48 ± 1.0 (IC50 mg/mL)			
BCB	0.23 ± 0.002 (IC50 mg/mL)			
FRAP	3.9 ± 0.5 P (µm Fe (II)/g)			
DPPH	147 ± 1.23 IC50 µg/mL	peel	Maceration	[50]
BCB	3 ± 0.05 IC50 µg/mL at 30 min			
Bovine brain peroxidation assay	2472 ± 4.19 IC50 µg/mL			
DPPH	72.00 ± 0.82% scavenging activity	juice	Maceration	[3]
TPC	309.08 ± 3.06 mgGAE/g			
DPPH	EC_50_ 102.9 µg/mL	leaves	Maceration	[38]
TPC	398.0 ± 3.2 mg/100 g FW	flowers	Exhaustive maceration	[27]
	401.6 ± 5.1 mg/100 g FW	leaves		
	181.3 ± 3.1 mg/100 g FW	immature mesocarp		
	262.6 ± 3.7 mg/100 g FW	immature endocarp		
	123.1 ± 6.5 mg/100 g FW	mature mesocarp		
	109.4 ± 2.9 mg/100 g FW	mature endocarp		
TFC	266.9 ± 7.2 mg QUE/100 g FW	flowers		
	97.5 ± 2.8 mg QUE/100 g FW	leaves		
	95.7 ± 3.2 mg QUE/100 g FW	immature mesocarp		
	64.9 ± 3.2 mg QUE/100 g FW	immature endocarp		
	43.1 ± 1.2 mg QUE/100 g FW	mature mesocarp		
	37.5 ± 1.6 mg QUE/100 g FW	mature endocarp		
DPPH	425.0 ± 2.95 µg Ascorbic acid/mL	flowers		
	502.0 ± 3.01 µg Ascorbic acid/mL	leaves		
	382.0 ± 2.45 µg Ascorbic acid/mL	immature mesocarp		
	>1000 µg Ascorbic acid/mL	immature endocarp		
	>1000 µg Ascorbic acid/mL	mature mesocarp		
	>1000 µg Ascorbic acid/mL	mature endocarp		
BCB	2.8 ± 0.002 µg/mL at 30 min	flowers		
	>100 µg/mL at 30 min	leaves		
	3.7 ± 0.007 µg/mL at 30 min	immature mesocarp		
	4.1 ± 0.009 µg/mL at 30 min	immature endocarp		
	36.6 ± 0.075 µg/mL at 30 min	mature mesocarp		
	3.5 ± 0.008 µg/mL at 30 min	mature endocarp		
TPC	227.45 mg GAE/100 g FW	peel	Maceration 70% MeOH	[26]
	88.76 mg GAE/100 g FW	pulp		
DPPH	IC50 22.79 μg GAE/ml	peel		
	IC50 54.74 μg GAE/mL	pulp		
TPC	2.52 ± 0.07 mg GAE/g	exocarp	UAE	[54]
	1.74 ± 0.02 mg GAE/g	mesocarp		
TFC	2.20 ± 0.26 mg QE/g	exocarp		
	1.50 ± 0.06 mg QE/g	mesocarp		
ABTS	55.8 ± 5.4% RSA	exocarp		
	52.0 ± 0.4% RSA	mesocarp		
	54.1 ± 0.2% RSA	EO	Hydro-distillation	
	3.1 ± 0.2% RSA	Hy		
DPPH	55.7 ± 1.20% RSA	exocarp	UAE	
	46.7 ± 0.82% RSA	mesocarp		
	26.4 ± 0.74% RSA	EO	Hydro-distillation	
	2.5 ± 0.3% RSA	Hy		
DPPH	77.2% RSA	EO	Hydro-distillation	[46]
TPC	2.74 ± 1.12 mg GAE/g	fructus	UAE	[35]
TFC	2.41 ± 2.03 mg QUE/g	fructus		
DPPH	1.48 ± 1.82 TE mM/g	fructus		
ABTS	0.92 ± 2.08 TE mM/g	fructus		
FRAP	0.38 ± 1.98 FeII mM/g	fructus		
TPC	31.60 ± 0.35 mg GAE/g	fructus	Maceration and UAE	[15]
TFC	15.38 ± 0.02 mg RE/g			
DPPH	EC_50_ 78.00% μg/mL			
DPPH	47.45% (3.2 mg/mL)	fructus	Maceration in 95% ethanol and 0.3 mol/L of NaOH solution overnight	[61]
ABTS	49.58% (3.2 mg/mL)			
DPPH	90.24% at 1.0 mg/mL	fructus	CPE	[41]
ORAC	928.64 µmol TE/g			
Hydroxyl RSA	81.5% at 0.8 mg/mL	fructus	Digestion	[45]
Superoxide anion radical scavenging activity	7.7 to 73.5% at 0.05 to 0.8 mg/mL			
TPC	25.8 ± 2.8 mg GAE/g of DW	by-products	Maceration 96% EtOH	[68]
DPPH	43.8 ± 0.3%			
**Antimicrobial, antiviral and antifungal activity**				
MTT	95% inhibition at 0.5 µg/µL on Madin Darby canine kidney (MDCK) cell line with Avian influenza A virus (H5N1	EO	Hydro-distillation	[69]
Agar diffusion assay	MIC: *C. albicans* 3 µg/mL, *B. subtilis* 25 µg/mL, *K. pneumonia* 25 µg/mL	fructus	Hydro-distillation	[41]
	Inibition zone (mm): *B. subtilis* 13, *B. cereus* 21, *S. aureus* 12, *K. pneumonia* 15, *C. albicans* 27, *A. niger* 11	leaves		
Plaque reduction assay	50% at 0.504 µg/µl	fructus		
	95% at 0.5 µg/µl	leaves		
Plate count analysis	*Saccharomyces cerevisiae:* 3 min at 500 ppm	fructus	Hydro-distillation	[44]
Plate count analysis	Bacteria survival: *E. coli* (600 ppm) 1 log decrease at day 3, *S. Enteritidis* (600 ppm) 3 log decrease at day 3, *L. monocytogenes* (600 ppm) 4 log decrease at day 3	fructus	Hydro-distillation	[70]
Disc diffusion test	Inibition zone (mm): mold growth on bread from 8.54 ± 1.27 mm to 15.26 ± 2.16 mm	flower and fructus	Hydro-distillation	[71]
	Inibition zone (mm): mold growth on bread > 90 mm	leaves	Hydro-distillation	
Agar diffusion assay	MIC (µL/mL)*: Lactobacillus curvatus*, *Weissella viridescens*, *Leuconostoc mesenteroides*, *Enterococcus faecium*, *Lactobacillus reuteri*, *Lactobacillus dextrinicus*, *Lactobacillus sakei*, and *Pediococcus dextrinicus* from 7.33 ± 0.57 to 9.00 ± 0.00	fructus	Hydro-distillation	[72]
Agar diffusion assay	MIC (mg/mL): Gram-positive from 0.625 to 1.25; Gram-negative bacteria 2.5	fructus	Hydro-distillation	[73]
Plate count analysis	MIC (mg/mL): *Gluconobacter cerinus*, *Dekkera bruxellensis*, *Candida zemplinina*, *Hanseniaspora uvarum*, *Pichia guilliermondii*, and *Zygosaccharomyces bailii* from 530 to 4240	fructus	Hydro-distillation	[74]
Oxford cup method	MIC (mg/mL): *Fusarium oxysporum* 9.38, *Fusarium solani* 12.05, and *Cylindrocarpon destructans* 8.44	fructus	Hydro-distillation	[75]
Plate count analysis	*Yersinia enterocolitica O9*, *Proteus* spp., *Klebsiella pneumoniae*, and *E. coli:* not effective	aerial parts	Hydro-distillation	[76]
Agar diffusion assay	MIC (mg/L)*: Staphylococcus aureus*, *Staphylococcus epidermidis*, *Escherichia coli*, *Listeria monocytogenes*, *Salmonella Enteritidis*, *Salmonella Typhimurium*, *Pseudomonas fragi*, *Saccharomyces cerevisiae*, and *Aspergillus niger* < 2000	fructus	Hydro-distillation	[77]
Agar diffusion assay	MIC (*v*/*v*%): *Escherichia coli*, *Pseudomonas Aeruginosa*, *Salmonella paratyphi B*, *Listeria monocytogenes*, *Staphylococcus aureus*, *Bacillus subtilis*, *Candida albicans*, and *Aspergillus flavus* from 1 and 4% *v/v*	peel	Hydro-distillation	[46]
Biofilm formation	Inhibition of biofilm formation (%): *Staphylococcus aureus* 100% at 0.75 mg/mL	fructus	Ultrasonic/microwave assisted hydro-distillation	[78]
	Inhibition of biofilm formation (%): *Staphylococcus aureus* 83l% at 0.75 mg/mL		Maceration	
Agar diffusion assay	Gold fingered citron MIC (mg/mL): *Bacillus subtilis*, *Streptococcus pneumoniae*, *Enterococcus faecalis*, *Escherichia coli*, and *Staphylococcus aureus* from 0.00 to 10.82 ± 02	fructus	ultrasonic	[79]
	Cantonese fingered citron MIC (mg/mL): *Bacillus subtilis*, *Streptococcus pneumoniae*, *Enterococcus faecalis*, *Escherichia coli*, and *Staphylococcus aureus* from 0.00 to 9.81 ± 0.20			
	Sichuan fingered citron MIC (mg/mL): *Bacillus subtilis*, *Streptococcus pneumoniae*, *Enterococcus faecalis*, *Escherichia coli*, and *Staphylococcus aureus* from 0.00 to 10.83 ± 0.24			
Agar diffusion assay	Sulphur nanoparticles MIC (µg/mL): *Listeria monocytogenes*, *Salmonella typhi*, *Chromobacterium violaceum*, *Fusarium oxysporum*, and *Aspergillus flavus* from 250 ± 1.21 to 700 ± 1.88	leaves	Hydro-distillation	[80]
	Aluminium oxide nanoparticles MIC (µg/mL): *Listeria monocytogenes*, *Salmonella typhi*, *Chromobacterium violaceum*, *Fusarium oxysporum*, and *Aspergillus flavus* from 150 ± 2.77 to 1000 ± 1.1			
Tetrazolium microplate Assay	Nanoemulsions MIC (μL/mL): *Escherichia coli*, *Bacillus subtilis*, and *Staphylococcus aureus* from 0.16 to >2.5	commercial EO	Commercial EO	[81]
Mycelial growth assay	Nanoemulsions mycelial growth inhibition (%): *Penicillium citrinum* and *Aspergillus niger* from 3.6 ± 0.6 to 27.0 ± 1.1			
Agar diffusion assay	ZnO nanoparticles inhibition zone (mm): *Streptomyces sannanesis*, *Bacillus subtilis*, *Pseudomonas aeruginosa*, *Salmonella enterica*, *Candida albicans*, and *Aspergillus niger* from 22 to 25	peel	Maceration	[82]
Agar diffusion assay	Ethyl acetate and ethanolic extract MIC (mg/mL): *Staphylococcus auricularis* not active, *Streptococcus mitis* not active, *Streptococcus pneumoniae* not active, *Klebsiella pneumoniae*, and *Escherichia coli* from 12.5 to 25.	peel	Reflex extraction	[83]
	MIC (mg/mL): *Staphylococcus auricularis*, *Streptococcus mitis*, *Streptococcus pneumoniae*, and *Klebsiella pneumoniae* from 1.5625 to 6.25; *Escherichia coli* not active	juice	Hand squeezing	
Agar diffusion assay	Zone of inhibition (mm): *Bacillus subtilis*, *Staphylococcus aureus*, *Enterococcus faecalis*, *Escherichia coli*, *Klebsiella pneumoniae*, *Pseudomonas aeruginosa*, *Proteus vulgaris*, *Aspergillus flavus*, *A. niger*, and *Candida albicans* from 0 to 23	root, leaf, bark, peel, pulp	Maceration	[84]
		juice	Hand squeezing	
Agar diffusion assay	*Bacillus subtilis*, *Staphylococcus aureus*, and *Escherichia coli*; *Klebsiella pneumonia* not active	juice	Hand squeezing	[85]
Pour plate	(At 2.0 mg/mL) MIC: *P. aeruginosa* 82.8%, *S. aureus* 100%	fructus	Maceration	[21]
Microtiter-plate	*E. coli*, *L. monocytegenes*, *P. carotovorum*, *Ps. aeruginosa*, and *S. aureus* MIC (mg/mL): 7–10	peel	Maceration	[34]
		pulp		
Adherence and invasion in HeLA cells	Adherence (50.8 to 91%), invasion (85.1 to 94.8%) in *C. jejuni strain*	by-products	Maceration	[68]
Motility	Inhibition *C. jejuni*: 35–50%	peles, seeds, bagasse	Maceration	[86]
Biofilm formation	Inhibition *C. jejuni*: 60–75%,			
Disk diffusion	*E. coli*, *K. pneumoniae*, *P. aeruginosa*, *Propionibacterium acnes*, *Salmonella typhi*, and fungi *Fusarium culmorum*, *F. oxysporum*, and *F. graminearum; z*one of inhibition:10–30 mm	fructus	Squeezing	[87]
Crystal violet staining	MIC = 1.25% (*v*/*v*)	fructus	Carbon-quantum-dots synthesis	[88]
**Cytotoxic activity**				
MTT	Growth inhibition 56.5 ± 3.6% 50 µg/mL	peel	Hydro-distillation	[89]
	Growth inhibition 30.2 ± 2.2% and 73.3 ± 2.6% cell death at 25 and 50 μg/mL			[90]
	EC_50_ 1.24 ± 0.42 EO EC_50_ 2.97 ± 0.07 Hy	fructus		[54]
	EC_50_ 1.76 ± 0.32	exocarp	Maceration and UAE	[54]
	50% growth inhibition at 7.80 and 9.50 μmol/L	peel	Semisynthesis	[91]
	IC_50_ from 60.5 to 80.0 μM	bark	Maceration	[37]
**Anti-inflammatory and analgesic activity**				
NO	56% after 12 h, 83% after 24 h at 0.063 mg/mL	fructus	Hydro-distillation	[77]
	IC_50_ 17.0 mg/mL	peel		[92]
	10.87–82.77% at 500 mg (60–300 min)			[24]
	250–500 mg at 30–120 min			
Clinical study	> than placebo in reduction in headache-attack intensity	juice	Syrup	[10]
**Hypoglycemic activity**				
*α*-amylase	IC_50_ 625 ± 8.53 µg/mL	peel	Maceration	[50]
	>1000 IC_50_ µg/mL	flowers		[27]
	438.5 ± 5.2 IC_50_ µg/mL	leaves		
	702.2 ± 5.7 IC_50_ µg/mL	immature mesocarp		
	702.2 ± 5.7 IC_50_ µg/mL	immature endocarp		
	707.4 ± 5.6 IC_50_ µg/mL	mature mesocarp		
	426.0 ± 4.4 IC_50_ µg/mL	mature endocarp		
*α* -glucosidase	>1000 IC_50_ µg/mL	flowers		
	777.8 ± 5.4 IC_50_ µg/mL	leaves		
	539.7 ± 6.4 IC_50_ µg/mL	immature mesocarp		
	472.9 ± 4.7 IC_50_ µg/mL	immature endocarp		
	633.1 ± 3.4 IC_50_ µg/mL	mature mesocarp		
	574.1 ± 5.8 IC_50_ µg/mL	mature endocarp		
Plasma glucose level	Glucose (mg/dL): from 213 (60 min) to 155 (120 min)	epicarp	Hydro-distillation	[53]
	Glucose (mg/dL): from 228 (60 min) to 216(120 min)	pulp		
	From glucose level (mg/dL) 106.8 ± 5.87 to 105.2 ± 8.35 (after 1 month) at 200 mg/kg/day; from glucose level (mg/dL) 109.3 ± 5.04 to 87.4 ± 6.30 (after 1 month) at 400 mg/kg/day	leaves	Maceration	[38]

## Data Availability

Not applicable.

## Abbreviations

ABTS	2,2′-Azino-bis (3-ethylbenzothiazoline-6-sulfonic acid)
APE	Alginate extract and pectin filler
COSY	Correlation spectroscopy
CPE	Continuous phase-transition extraction
C-RDI	Contribution reference daily intake
DLA	Dalton’s lymphoma ascites
DPPH	2,2-Diphenyl-1-picrylhydrazyl
DW	Dry weight
EC_50_	Half maximal effective concentration
EAC	Ehrlich’s ascites carcinoma
EtOH	Ethanol
EI-MS	Electron ionization–mass spectrometry
EO	Essential oil
ERK	Extracellular signal-regulated kinase
FW	FW
GAE	Gallic acid equivalent
GC–MS	Gas chromatography–mass spectrometry
GC–MS–SPME	Gas chromatography–mass spectrometry–solid-phase microextraction
HeLa	Henrietta Lacks
HL60	Human leukemia cell line
HMQC	Heteronuclear multiple quantum coherence
HMBC	Heteronuclear multiple bond coherence
HPGPC	High-performance gel-permeation chromatography
HPLC	High-performance liquid chromatography
HPLC–PDA–MS	High-performance liquid chromatography–photodiode array–mass spectrometry
HPLC–QTOF–MS	High-performance liquid chromatography–quadruple time of flight–mass spectrometry
HRGC–MS	High-resolution gas chromatography–mass spectrometry
HR–ESI–MS	High-resolution–electrospray ionization–mass spectrometry
HR–EI–MS	High-resolution–electron ionization–mass spectrometry
HR–MAS–NMR	High-resolution–magic angle sinning–nuclear magnetic resonance
HY	Hydrolat
IC_50_	Half-maximal inhibitory concentration
IkB-*α:*	Nuclear factor of kappa light polypeptide gene enhancer in B-cells inhibitor, alpha
IL-6	Interleukin 6
IL-1β	Interleukin 1*β*
IL-10	Interleukin 10
JNK	c-Jun N-terminal kinase
LPS	Lipopolysaccharide
MAPKs	Mitogen-activated protein kinase
MeOH	Methanol
MIC	Minimum inhibitory concentration
NF-kB	Nuclear factor kappa-light-chain-enhancer of activated B cells
NO	Nitric oxide
NOESY	Nuclear Overhauser effect spectroscopy
PC-3	Human prostate adenocarcinoma cell line
PLE	Pressure liquid extraction
RAW 264.7	Macrophage cell line
RSA	Radical-scavenging activity
SARS-CoV-2	Severe Acute Respiratory Syndrome Coronavirus 2
SCF–CO2	Super-critical fluid–carbon bioxide
SH-SY5Y	Human neuroblastoma cell line
TFC	Total flavonoid content
TNF-α	Tumor necrosis factor alpha
TPC	Total phenolic content
UV	Ultraviolet
UAHD	Hydro-distillation ultrasound-assisted extraction
UAE	Ultrasound-assisted extraction
UHPLC–QTOF–IMS	Ultra-performance liquid chromatography–quadruple time of flight–mass spectrometry
Var.	Variety

## References

[1] Ramadugu C., Keremane M.L., Hu X., Karp D., Federici C.T., Kahn T., Roose M.L., Lee R.F. (2015). Genetic analysis of citron (*Citrus medica* L.) using simple sequence repeats and single nucleotide polymorphisms. Sci. Hortic..

[2] Hayat K. (2014). Citrus molecular phylogeny antioxidant properties and medicinal uses. Nova. Sci..

[3] Dey P., Hoque M., Islam M., Monalisa K. (2021). Kinetics of antioxidants degradation and quality changes of citron (*Citrus medica*) fruit juice concentrate during storage. J. Microbiol. Biotechnol. Food Sci..

[4] Pieroni A. (2000). Medicinal plants and food medicines in the folk traditions of the upper Lucca Province, Italy. J. Ethnopharmacol..

[5] Secundus C.P. (1865). Naturalis Historiae, Libri XXXVII.

[6] Pieroni A., Quave C.L., Villanelli M.L., Mangino P., Sabbatini G., Santini L., Boccetti T., Profili M., Ciccioli T., Rampa L.G. (2004). Ethnopharmacognostic survey on the natural ingredients used in folk cosmetics, cosmeceuticals and remedies for healing skin diseases in the inland Marches, Central-Eastern Italy. J. Ethnopharmacol..

[7] Sahoo C.R., Sahoo J., Mahapatra M., Lenka D., Sahu P.K., Dehury B., Padhy R.N., Paidesetty S.K. (2021). Coumarin derivatives as promising antibacterial agent (s). Arab. J. Chem..

[8] Haridas M., Sasidhar V., Nath P., Abhithaj J., Sabu A., Rammanohar P. (2021). Compounds of *Citrus medica* and Zingiber officinale for COVID-19 inhibition: In silico evidence for cues from Ayurveda. Future J. Pharm. Sci..

[9] Zarshenas M.M., Mahmoodian R., Moein M. (2015). Colorimetric determination of flavonoids in *Citrus medica* peel traditional medicinal oil. Int. J. Pharmacogn. Phytochem. Res..

[10] Jafarpour M., Yousefi G., Hamedi A., Shariat A., Salehi A., Heydari M. (2016). Effect of a traditional syrup from *Citrus medica* L. fruit juice on migraine headache: A randomized double blind placebo controlled clinical trial. J. Ethnopharmacol..

[11] Kalariya M., Prajapati R., Chavda J. (2019). Pharmacological potential of *Citrus medica*: A review. Pharma Sci. Monit..

[12] Karp D., Hu X. (2018). The citron (*Citrus medica* L.) in China. Hortic. Rev..

[13] Cumpston M., Li T., Page M.J., Chandler J., Welch V.A., Higgins J.P., Thomas J. (2019). Updated guidance for trusted systematic reviews: A new edition of the Cochrane Handbook for Systematic Reviews of Interventions. Cochrane Database Syst. Rev..

[14] Ponticelli M., Lela L., Moles M., Mangieri C., Bisaccia D., Faraone I., Falabella R., Milella L. (2022). The healing bitterness of Gentiana lutea L., phytochemistry and biological activities: A systematic review. Phytochemistry.

[15] Dadwal V., Joshi R., Gupta M. (2022). A comparative metabolomic investigation in fruit sections of *Citrus medica* L. and *Citrus maxima* L. detecting potential bioactive metabolites using UHPLC-QTOF-IMS. Food Res. Int..

[16] Hasan M.M., Roy P., Alam M., Hoque M.M., Zzaman W. (2022). Antimicrobial activity of peels and physicochemical properties of juice prepared from indigenous citrus fruits of Sylhet region, Bangladesh. Heliyon.

[17] Mahdi A.A., Al-Ansi W., Ahmed M.I., Xiaoyun C., Mohammed J.K., Sulieman A.A., Mushtaq B.S., Harimana Y., Wang H. (2020). Microwave assisted extraction of the bioactive compounds from peel/pulp of *Citrus medica* L. var. sarcodactylis swingle along with its nutritional profiling. J. Food Meas. Charact..

[18] Khomdram S., Barthakur S., Devi G.S. (2014). Biochemical and molecular analysis of wild endemic fruits of the Manipur region of India. Int. J. Fruit Sci..

[19] Madhavi N., Jyothi B. (2016). Colorimetric determination of vitamin C in fresh and dilute fruit juices and effect of thermal exposure on concentration at various stages. Int. J. Pharma Bio. Sci..

[20] Pallavi M., Ck R., Krishna V., Parveen S. (2017). Quantitative phytochemical analysis and antioxidant activities of some citrus fruits of South India. Asian J. Pharm. Clin. Res..

[21] Wang E., Li Y., Maguy B.L., Lou Z., Wang H., Zhao W., Chen X. (2019). Separation and enrichment of phenolics improved the antibiofilm and antibacterial activity of the fractions from *Citrus medica* L. var. sarcodactylis in vitro and in tofu. Food Chem..

[22] Mucci A., Parenti F., Righi V., Schenetti L. (2013). Citron and lemon under the lens of HR-MAS NMR spectroscopy. Food Chem..

[23] Bellavite P., Donzelli A. (2020). Hesperidin and SARS-CoV-2: New light on the healthy function of citrus fruits. Antioxidants.

[24] Malleshappa P., Kumaran R.C., Venkatarangaiah K., Parveen S. (2018). Peels of citrus fruits: A potential source of anti-inflammatory and anti-nociceptive agents. Pharmacogn. J..

[25] Adham A.N. (2015). Comparative antimicrobrial activity of peel and juice extract of citrus fruits growing in Kurdistan/Iraq. Am. J. Microbiol. Re..

[26] Taghvaeefard N., Ghani A., Hosseinifarahi M. (2021). Comparative study of phytochemical profile and antioxidant activity of flavedo from two Iranian citron fruit (*Citrus medica* L.). J. Food Meas. Charact..

[27] Menichini F., Loizzo M.R., Bonesi M., Conforti F., De Luca D., Statti G.A., de Cindio B., Menichini F., Tundis R. (2011). Phytochemical profile, antioxidant, anti-inflammatory and hypoglycemic potential of hydroalcoholic extracts from *Citrus medica* L. cv Diamante flowers, leaves and fruits at two maturity stages. Food Chem. Toxicol..

[28] Adham A.N. (2015). Qualitative and quantitative estimation of hesperidin in peel and juice of citrus fruits by RP-HPLC method growing in Kurdistan region/Iraq. Int. J. Pharm. Sci. Rev. Res..

[29] Choi I.-W., Choi S.-Y., Ji J.-R. (2009). Flavonoids and functional properties of germinated citron (Citrus junos Sieb. ex TANAKA) shoots. Food Sci. Biotechnol..

[30] Tahir T., Ashfaq M., Shahzad M.I., Bukhari S.T. (2019). Recent Approaches in the Extraction of Citrus Metabolites. Curr. Biotechnol..

[31] Govindarajan D.K., Viswalingam N., Meganathan Y., Devaraj B.S., Sivamani S., Sivarajasekar N. (2022). Response surface methodology optimization for extraction of pectin from waste rinds of *Citrus medica*. AIP Conf. Proc..

[32] Ma Q.-G., Wei R.-R., Yang M., Huang X.-Y., Wang F., Dong J.-H., Sang Z.-P. (2021). Isolation and characterization of neolignan derivatives with hepatoprotective and neuroprotective activities from the fruits of *Citrus medica* L. var. Sarcodactylis Swingle. Bioorg. Chem..

[33] Menichini F., Tundis R., Loizzo M.R., Bonesi M., D’Angelo D., Lombardi P., Mastellone V. (2016). *Citrus medica* L. cv Diamante (Rutaceae) peel extract improves glycaemic status of Zucker diabetic fatty (ZDF) rats and protects against oxidative stress. J. Enzyme Inhib..

[34] Fratianni F., Cozzolino A., De Feo V., Coppola R., Ombra M.N., Nazzaro F. (2019). Polyphenols, antioxidant, antibacterial, and biofilm inhibitory activities of peel and pulp of *Citrus medica* L., Citrus bergamia, and *Citrus medica* cv. Salò cultivated in southern Italy. Molecules.

[35] Zhao P., Duan L., Guo L., Dou L.-L., Dong X., Zhou P., Li P., Liu E.-H. (2015). Chemical and biological comparison of the fruit extracts of Citrus wilsonii Tanaka and *Citrus medica* L.. Food Chem..

[36] Chan Y.-Y., Li C.-H., Shen Y.-C., Wu T.-S. (2010). Anti-inflammatory principles from the stem and root barks of *Citrus medica*. Chem. Pharm. Bull..

[37] Fomani M., Happi E.N., Francois-Hugues P., Lallemand M.-C., Waffo A.F.K., Wansi J.D. (2015). A prenylated acridone alkaloid and ferulate xanthone from barks of *Citrus medica* (Rutaceae). ZNB.

[38] Hetta M., El-Alfy T., Yassin N., Abdel-Rahman R., Kadry E. (2013). Phytochemical and antihyperglycemic studies on *Citrus medica* L. leaves (Etrog) growing in Egypt. Int. J. Pharmacogn. Phytochem. Res..

[39] Roowi S., Crozier A. (2011). Flavonoids in tropical citrus species. J. Agric. Food Chem..

[40] Chu J., Li S.-L., Yin Z.-Q., Ye W.-C., Zhang Q.-W. (2012). Simultaneous quantification of coumarins, flavonoids and limonoids in Fructus Citri Sarcodactylis by high performance liquid chromatography coupled with diode array detector. J. Pharm. Biomed. Anal..

[41] Luo X., Wang J., Chen H., Zhou A., Song M., Zhong Q., Chen H., Cao Y. (2020). Identification of flavoanoids from finger citron and evaluation on their antioxidative and antiaging activities. Front. Nutr..

[42] Mondal M., Saha S., Sarkar C., Hossen M.S., Hossain M.S., Khalipha A.B.R., Islam M.F., Wahed T.B., Islam M.T., Rauf A. (2021). Role of *Citrus medica* L. Fruits Extract in Combatting the Hematological and Hepatic Toxic Effects of Carbofuran. Chem. Res. Toxicol..

[43] Chan Y.-Y., Hwang T.-L., Kuo P.-C., Hung H.-Y., Wu T.-S. (2017). Constituents of the Fruits of *Citrus medica* L. var. sarcodactylis and the Effect of 6, 7-Dimethoxy-coumarin on Superoxide Anion Formation and Elastase Release. Molecules.

[44] Belletti N., Kamdem S.S., Patrignani F., Lanciotti R., Covelli A., Gardini F. (2007). Antimicrobial activity of aroma compounds against Saccharomyces cerevisiae and improvement of microbiological stability of soft drinks as assessed by logistic regression. Appl. Environ. Microbiol..

[45] Wu Z., Li H., Yang Y., Zhan Y., Tu D. (2013). Variation in the components and antioxidant activity of *Citrus medica* L. var. sarcodactylis essential oils at different stages of maturity. Ind. Crops Prod..

[46] Guo J.-J., Gao Z.-P., Xia J.-L., Ritenour M.A., Li G.-Y., Shan Y. (2018). Comparative analysis of chemical composition, antimicrobial and antioxidant activity of citrus essential oils from the main cultivated varieties in China. LWT.

[47] Jing L., Lei Z., Zhang G., Pilon A.C., Huhman D.V., Xie R., Xi W., Zhou Z., Sumner L.W. (2015). Metabolite profiles of essential oils in citrus peels and their taxonomic implications. Metabolomics.

[48] Poiana M., Sicari V., Mincione B. (1998). A comparison between the chemical composition of the oil, solvent extract and supercritical carbon dioxide extract of *Citrus medica* cv. Diamante. J. Essent. Oil Res..

[49] Gabriele B., Fazio A., Dugo P., Costa R., Mondello L. (2009). Essential oil composition of *Citrus medica* L. Cv. Diamante (Diamante citron) determined after using different extraction methods. J. Sep. Sci..

[50] Conforti F., Statti G.A., Tundis R., Loizzo M.R., Menichini F. (2007). In vitro activities of *Citrus medica* L. cv. Diamante (Diamante citron) relevant to treatment of diabetes and Alzheimer’s disease. Phytother. Res..

[51] Xing C., Qin C., Li X., Zhang F., Linhardt R.J., Sun P., Zhang A. (2019). Chemical composition and biological activities of essential oil isolated by HS-SPME and UAHD from fruits of bergamot. LWT.

[52] Wei L., Yu X., Li H., Zhu M., Pu D., Lu Q., Bao Y., Zu Y. (2023). Optimization of solvent-free microwave extraction of essential oil from the fresh peel of *Citrus medica* L. var. arcodactylis Swingle by response surface methodology, chemical composition and activity characterization. Sci. Hortic..

[53] Peng C.-H., Ker Y.-B., Weng C.-F., Peng C.-C., Huang C.-N., Lin L.-Y., Peng R.Y. (2009). Insulin secretagogue bioactivity of finger citron fruit (*Citrus medica* L. var. Sarcodactylis Hort, Rutaceae). J. Agric. Food Chem..

[54] Vitalini S., Iriti M., Ovidi E., Laghezza Masci V., Tiezzi A., Garzoli S. (2022). Detection of Volatiles by HS-SPME-GC/MS and Biological Effect Evaluation of Buddha’s Hand Fruit. Molecules.

[55] Fanciullino A.-L., Dhuique-Mayer C., Luro F., Casanova J., Morillon R., Ollitrault P. (2006). Carotenoid diversity in cultivated citrus is highly influenced by genetic factors. J. Agric. Food Chem..

[56] Lim J.Y., Yoon H.-S., Kim K.-Y., Kim K.-S., Noh J.G., Song I.G. (2010). Optimum conditions for the enzymatic hydrolysis of citron waste juice using response surface methodology (RSM). Food Sci. Biotechnol..

[57] Belletti N., Ndagijimana M., Sisto C., Guerzoni M.E., Lanciotti R., Gardini F. (2004). Evaluation of the antimicrobial activity of citrus essences on Saccharomyces cerevisiae. J. Agric. Food Chem..

[58] Kim K.-N., Ko Y.-J., Yang H.-M., Ham Y.-M., Roh S.W., Jeon Y.-J., Ahn G., Kang M.-C., Yoon W.-J., Kim D. (2013). Anti-inflammatory effect of essential oil and its constituents from fingered citron (*Citrus medica* L. var. sarcodactylis) through blocking JNK, ERK and NF-κB signaling pathways in LPS-activated RAW 264.7 cells. Food Chem. Toxicol..

[59] Gancel A.L., Ollitrault P., Froelicher Y., Tomi F., Jacquemond C., Luro F., Brillouet J.M. (2005). Citrus somatic allotetraploid hybrids exhibit a differential reduction of leaf sesquiterpenoid biosynthesis compared with their parents. Flavour. Fragr. J..

[60] Wang S.F., Ju Y., Chen X.G., De Hu Z. (2003). Separation and determination of coumarins in the root bark of three citrus plants by micellar electrokinetic capillary chromatography. Planta Med..

[61] Peng B., Yang J., Huang W., Peng D., Bi S., Song L., Wen Y., Zhu J., Chen Y., Yu R. (2019). Structural characterization and immunoregulatory activity of a novel heteropolysaccharide from bergamot (*Citrus medica* L. var. sarcodactylis) by alkali extraction. Ind. Crops Prod..

[62] Peng B., Luo Y., Hu X., Song L., Yang J., Zhu J., Wen Y., Yu R. (2019). Isolation, structural characterization, and immunostimulatory activity of a new water-soluble polysaccharide and its sulfated derivative from *Citrus medica* L. var. sarcodactylis. Int. J. Biol. Macromol..

[63] Gao Y., Peng B., Xu Y., Yang J.-n., Song L.-y., Bi S.-x., Chen Y., Zhu J.-h., Wen Y., Yu R.-m. (2019). Structural characterization and immunoregulatory activity of a new polysaccharide from *Citrus medica* L. var. sarcodactylis. RSC Adv..

[64] He Z., Liang F., Zhang Y., Pan Y. (2014). Water-soluble polysaccharides from finger citron fruits (*Citrus medica* L. var. sarcodactylis). Carbohydr. Res..

[65] Chang X., Shen C.-Y., Jiang J.-G. (2021). Structural characterization of novel arabinoxylan and galactoarabinan from citron with potential antitumor and immunostimulatory activities. Carbohydr. Polym..

[66] Luo B., Lv J., Li K., Liao P., Chen P. (2022). Structural Characterization and Anti-inflammatory Activity of a Galactorhamnan Polysaccharide From *Citrus medica* L. var. sarcodactylis. Front. Nutr..

[67] Wu Z., Li H., Tu D., Yang Y., Zhan Y. (2013). Extraction optimization, preliminary characterization, and in vitro antioxidant activities of crude polysaccharides from finger citron. Ind. Crops Prod..

[68] Castillo S., Dávila-Aviña J., Heredia N., Garcia S. (2017). Antioxidant activity and influence of Citrus byproduct extracts on adherence and invasion of Campylobacter jejuni and on the relative expression of cadF and ciaB. Food Sci. Biotechnol..

[69] El Hawary S.S.E., Hashim F.A., Ibrahim N.A., Matloub A.A., ElSayed A.M., Farid M.A., Kutkat O., El-Abd E.A., Ali M., Shata H.M. (2022). A Comparative study of Chemical Compositions, Antimicrobial and Antiviral Activities of Essential Oils for *Citrus medica* var. sarcodactylis Swingle Fruits and Leaves along with Limonia acidissima L. Leaves. Egypt J. Chem..

[70] Belletti N., Lanciotti R., Patrignani F., Gardini F. (2008). Antimicrobial efficacy of citron essential oil on spoilage and pathogenic microorganisms in fruit-based salads. J. Food Sci..

[71] Wu K., Jin R., Bao X., Yu G., Yi F. (2021). Potential roles of essential oils from the flower, fruit and leaf of *Citrus medica* L. var. sarcodactylis in preventing spoilage of Chinese steamed bread. Food Biosci..

[72] Khorsandi A., Ziaee E., Shad E., Razmjooei M., Eskandari M.H., Aminlari M. (2018). Antibacterial effect of essential oils against spoilage bacteria from vacuum-packed cooked cured sausages. J. Food Prot..

[73] Li Z.-H., Cai M., Liu Y.-S., Sun P.-L., Luo S.-L. (2019). Antibacterial activity and mechanisms of essential oil from *Citrus medica* L. var. sarcodactylis. Molecules.

[74] Mitropoulou G., Nikolaou A., Santarmaki V., Sgouros G., Kourkoutas Y. (2020). *Citrus medica* and Cinnamomum zeylanicum essential oils as potential biopreservatives against spoilage in low alcohol wine products. Foods.

[75] Chen W.F., Wong L.L., Zhang X., Zhou F.X., Xia F., Tang L.P., Li X. (2019). New 4, 5-seco-20 (10→5)-abeo-Abietane Diterpenoids with Anti-Inflammatory Activity from Isodon lophanthoides var. graciliflorus (Benth.) H. Hara. Chem. Biodivers..

[76] Al-Mariri A., Safi M. (2014). In vitro antibacterial activity of several plant extracts and oils against some gram-negative bacteria. Iran. J. Med. Sci..

[77] Mitropoulou G., Fitsiou E., Spyridopoulou K., Tiptiri-Kourpeti A., Bardouki H., Vamvakias M., Panas P., Chlichlia K., Pappa A., Kourkoutas Y. (2017). *Citrus medica* essential oil exhibits significant antimicrobial and antiproliferative activity. LWT.

[78] Zhang H., Lou Z., Chen X., Cui Y., Wang H., Kou X., Ma C. (2019). Effect of simultaneous ultrasonic and microwave assisted hydrodistillation on the yield, composition, antibacterial and antibiofilm activity of essential oils from *Citrus medica* L. var. sarcodactylis. J. Food Eng..

[79] Wang F., You H., Guo Y., Wei Y., Xia P., Yang Z., Ren M., Guo H., Han R., Yang D. (2020). Essential oils from three kinds of fingered citrons and their antibacterial activities. Ind. Crops Prod..

[80] Suryavanshi P., Pandit R., Gade A., Derita M., Zachino S., Rai M. (2017). Colletotrichum sp.-mediated synthesis of sulphur and aluminium oxide nanoparticles and its in vitro activity against selected food-borne pathogens. LWT.

[81] Li Z.-H., Cai M., Liu Y.-s., Sun P.-L. (2018). Development of finger citron (*Citrus medica* L. var. sarcodactylis) essential oil loaded nanoemulsion and its antimicrobial activity. Food Control.

[82] Keerthana P., Vijayakumar S., Vidhya E., Punitha V., Nilavukkarasi M., Praseetha P. (2021). Biogenesis of ZnO nanoparticles for revolutionizing agriculture: A step towards anti-infection and growth promotion in plants. Ind. Crops Prod..

[83] Adham A.N. (2015). Phytochemical analysis and evaluation antibacterial activity of *Citrus medica* peel and juice growing in Kurdistan/Iraq. J. Appl. Pharm. Sci..

[84] Sah A.N., Juyal V., Melkani A.B. (2011). Antimicrobial activity of six different parts of the plant *Citrus medica* Linn. Pharmacogn. J..

[85] Sharma R., Verma S., Rana S., Rana A. (2018). Rapid screening and quantification of major organic acids in citrus fruits and their bioactivity studies. J. Food Sci. Technol..

[86] Castillo S., Heredia N., Arechiga-Carvajal E., García S. (2014). Citrus extracts as inhibitors of quorum sensing, biofilm formation and motility of Campylobacter jejuni. Food Biotechnol..

[87] Shende S., Ingle A.P., Gade A., Rai M. (2015). Green synthesis of copper nanoparticles by *Citrus medica* Linn.(Idilimbu) juice and its antimicrobial activity. World J. Microbiol. Biotechnol..

[88] Selvaraju N., Ganesh P.S., Palrasu V., Venugopal G., Mariappan V. (2022). Evaluation of Antimicrobial and Antibiofilm Activity of *Citrus medica* Fruit Juice Based Carbon Dots against Pseudomonas aeruginosa. ACS Omega.

[89] Nair A., Kurup Sr R., Nair A.S., Baby S. (2018). Citrus peels prevent cancer. Phytomedicine.

[90] Ajikumaran N.S., Rajani Kurup S., Akhila S., Sabulal B. (2018). Citrus peels prevent cancer. Phytomedicine.

[91] Dahiya R., Kumar A. (2008). Synthetic and biological studies on a cyclopolypeptide of plant origin. J. Zhejiang Univ. Sci. B.

[92] Tundis R., Calabria U., Bonesi M., Calabria U., Calabria U. (2011). Chemical composition and bioactivity of *Citrus medica* L. cv. Diamante essential oil obtained by hydrodistillation. Nat. Prod. Res..

[93] Singh B., Singh J.P., Kaur A., Singh N. (2020). Phenolic composition, antioxidant potential and health benefits of citrus peel. Food Res. Int..

[94] Chen W., Zheng J., Li Y., Guo W. (2012). Effects of high temperature on photosynthesis, chlorophyll fluorescence, chloroplast ultrastructure, and antioxidant activities in fingered citron. Russ. J. Plant Physiol..

[95] Ebrahimi Y., Hasanvand A., Valibeik A., Ebrahimi F., Abbaszadeh S. (2019). Natural antioxidants and medicinal plants effective on hyperlipidemia. Res. J. Pharm. Technol..

[96] Jayaprakasha G., Patil B.S. (2007). In vitro evaluation of the antioxidant activities in fruit extracts from citron and blood orange. Food Chem..

[97] Pallavi M., Ramesh C., Siddesha J., Krishna V., Kavitha G., Nethravathi A., Parveen S., KM A.K. (2021). Anti-hyperlipidemic effects of citrus fruit peel extracts against high fat diet-induced hyperlipidemia in rats. Int. J. Res. Pharm. Sci..

[98] Ghani A., Taghvaeefard N., Hosseinifarahi M., Dakhlaoui S., Msaada K. (2022). Essential oil composition and antioxidant activity of citron fruit (*Citrus medica* var. macrocarpa Risso.) peel as relation to ripening stages. Int. J. Environ. Health Res..

[99] Dadwal V., Joshi R., Gupta M. (2021). Formulation, characterization and in vitro digestion of polysaccharide reinforced Ca-alginate microbeads encapsulating *Citrus medica* L. phenolics. LTW.

[100] Wu Z. (2015). Effect of different drying methods on chemical composition and bioactivity of finger citron polysaccharides. Int. J. Biol. Macromol..

[101] Mengsa H., Kun X., Pei L., Jun L. (2022). Five Rutaceae family ethanol extracts alleviate H_2_O_2_ and LPS-induced inflammation via NF-κB and JAK-STAT3 pathway in HaCaT cells. Chin. J. Nat. Med..

[102] Hanafy S.M., Abd El-Shafea Y.M., Saleh W.D., Fathy H.M. (2021). Chemical profiling, in vitro antimicrobial and antioxidant activities of pomegranate, orange and banana peel-extracts against pathogenic microorganisms. J. Genet. Eng. Biotechnol..

[103] Herman A., Tambor K., Herman A. (2016). Linalool affects the antimicrobial efficacy of essential oils. Curr. Microbiol..

[104] Nagy M.M., Al-Mahdy D.A., Abd El Aziz O.M., Kandil A.M., Tantawy M.A., El Alfy T.S. (2018). Chemical composition and antiviral activity of essential oils from Citrus reshni hort. ex Tanaka (Cleopatra mandarin) cultivated in Egypt. J. Essent. Oil-Bear. Plants.

[105] Punitha V., Vijayakumar S., Nilavukkarasi M., Vidhya E., Praseetha P. (2022). Fruit peels that unlock curative potential: Determination of biomedical application and bioactive compounds. S. Afr. J. Bot..

[106] Thi T.U.D., Nguyen T.T., Thi Y.D., Thi K.H.T., Phan B.T., Pham K.N. (2020). Green synthesis of ZnO nanoparticles using orange fruit peel extract for antibacterial activities. RSC Adv..

[107] Bray F., Laversanne M., Weiderpass E., Soerjomataram I. (2021). The ever-increasing importance of cancer as a leading cause of premature death worldwide. Cancer.

[108] Nyrop K.A., Damone E.M., Deal A.M., Wheeler S.B., Charlot M., Reeve B.B., Basch E., Shachar S.S., Carey L.A., Reeder-Hayes K.E. (2022). Patient-reported treatment toxicity and adverse events in Black and White women receiving chemotherapy for early breast cancer. Breast Cancer Res. Treat..

[109] Cirmi S., Maugeri A., Ferlazzo N., Gangemi S., Calapai G., Schumacher U., Navarra M. (2017). Anticancer potential of citrus juices and their extracts: A systematic review of both preclinical and clinical studies. Front. Pharmacol..

[110] Aliberti L., Caputo L., De Feo V., De Martino L., Nazzaro F., Souza L.F. (2016). Chemical composition and in vitro antimicrobial, cytotoxic, and central nervous system activities of the essential oils of *Citrus medica* L. cv.‘Liscia’and *C. medica* cv.‘Rugosa’cultivated in Southern Italy. Molecules.

[111] Rawat A., Reddy A.V.B. (2022). Recent advances on anticancer activity of coumarin derivatives. Eur. J. Med. Chem..

[112] Xiao S., Liu W., Bi J., Liu S., Zhao H., Gong N., Xing D., Gao H., Gong M. (2018). Anti-inflammatory effect of hesperidin enhances chondrogenesis of human mesenchymal stem cells for cartilage tissue repair. J. Inflamm..

[113] Sood S., Bansal S., Muthuraman A., Gill N., Bali M. (2009). Therapeutic potential of *Citrus medica* L. peel extract in carrageenan induced inflammatory pain in rat. J. Med. Plant Res..

[114] Marouf B.H., Hussain S.A., Ali Z.S., Ahmmad R.S. (2018). Resveratrol supplementation reduces pain and inflammation in knee osteoarthritis patients treated with meloxicam: A randomized placebo-controlled study. J. Med. Food.

[115] Awosika T.O., Aluko R.E. (2019). Inhibition of the in vitro activities of α-amylase, α-glucosidase and pancreatic lipase by yellow field pea (*Pisum sativum* L.) protein hydrolysates. Int. J. Food Sci. Technol..

[116] Tolmie M., Bester M.J., Apostolides Z. (2021). Inhibition of α-glucosidase and α-amylase by herbal compounds for the treatment of type 2 diabetes: A validation of in silico reverse docking with in vitro enzyme assays. J. Diabetes.

[117] Abdou H.M., Hamaad F.A., Ali E.Y., Ghoneum M.H. (2022). Antidiabetic efficacy of Trifolium alexandrinum extracts hesperetin and quercetin in ameliorating carbohydrate metabolism and activating IR and AMPK signaling in the pancreatic tissues of diabetic rats. Biomed. Pharmacother..

[118] Wahyuono R.A., Hesse J., Hipler U.-C., Elsner P., Böhm V. (2017). In vitro lipophilic antioxidant capacity, antidiabetic and antibacterial activity of citrus fruits extracts from Aceh, Indonesia. Antioxidants.

[119] Dehghan M., Fathinejad F., Farzaei M.H., Barzegari E. (2022). In silico unraveling of molecular anti-neurodegenerative profile of *Citrus medica* flavonoids against novel pharmaceutical targets. Chem. Pap..

[120] Figueiredo R.T., Bittencourt V.C.B., Lopes L.C.L., Sassaki G., Barreto-Bergter E. (2012). Toll-like receptors (TLR2 and TLR4) recognize polysaccharides of Pseudallescheria boydii cell wall. Carbohydr. Res..

